# Ultrasound-assisted continuous aqueous synthesis of sulfonate, imidazolate, and carboxylate MOFs with high space time yield

**DOI:** 10.1038/s42004-025-01548-5

**Published:** 2025-05-16

**Authors:** Chao Sun, Sang T. Pham, Sarah L. Boyall, Ben Douglas, Andrew J. Britton, Stuart Micklethwaite, Thomas W. Chamberlain, Maximilian O. Besenhard, Rik Drummond-Brydson, Ke-Jun Wu, Sean M. Collins

**Affiliations:** 1 School of Chemical and Process Engineering, University of Leeds, Leeds, UK; 2 School of Chemistry, University of Leeds, Leeds, UK; 3 Bragg Centre for Materials Research, University of Leeds, Leeds, UK; 4 Department of Chemical Engineering, University College London, London, UK; 5 Zhejiang Provincial Key Laboratory of Advanced Chemical Engineering Manufacture Technology, College of Chemical and Biological Engineering, Zhejiang University, Hangzhou, China

**Keywords:** Metal-organic frameworks, Chemical engineering, Flow chemistry

## Abstract

The boom in metal–organic frameworks (MOFs) for applications from chemical separations and gas storage to membranes for energy conversion and storage has stimulated interest in scalable MOF production methods. Combining the increased heat and mass transfer of flow reactors with the enhanced mixing and nucleation rates of sono-chemical synthesis, we developed an ultrasound-assisted two-phase flow platform for the aqueous synthesis of MOFs spanning three ligand chemistries, sulfonate Ca-NDS (water), imidazolate ZIF-8, and carboxylate UiO-66-NH_2_. We show that this reactor does not foul, facilitating continuous operation at an STY of 3.4 × 10^4^ (±1 × 10^3^) kg m^−3^ day^−1^ of proton-conducting Ca-NDS (water). ZIF-8 and UiO-66-NH_2_ MOFs prepared in ultrasound-assisted flow with smaller, uniform particle sizes exhibited matched or superior gas sorption to those made in batch. These results highlight the potential of ultrasound-assisted flow synthesis for MOFs, offering enhanced nucleation alongside process intensification, and paving the way for more efficient MOF production.

## Abstract (web summary)

The surge in metal–organic frameworks for diverse applications has stimulated significant interest in their sustainable and scalable production. Here, the authors develop an ultrasound-assisted two-phase flow reaction platform for the continuous aqueous synthesis of MOFs with sulfonate, imidazolate, and carboxylate ligand chemistries, achieving enhanced nucleation and process efficiency.

## Introduction

The scalable production of metal–organic frameworks (MOFs) underpins the technological realization of the promise that they have shown in applications ranging from gas storage^1^, chemical separations^2^, biomedical applications^3^, and catalysis^4^ to proton conduction^5^. Intensification of MOF production, however, risks deleterious environmental impact without green chemical synthesis implemented in scalable reactor designs. The vast majority of MOFs have been reported using syntheses lasting several hours or days and using toxic solvents, impeding scale-up and sustainable production^6,7^. These synthetic approaches, frequently employing dimethylformamide (DMF) or diethylformamide (DEF) as the solvent or in the solvent system, support diverse metal-based secondary building units and diverse chemistries of organic ligands^8^, such as sulfonates for proton conduction MOFs^9^, carboxylates for UiO^10^, HKUST^11^, MIL^12^, and IRMOF^13^ families, and imidazolates for the zeolitic imidazolate frameworks^14^. Although a number of aqueous syntheses have been reported recently, the solvent system alone addresses only part of the challenge. Minimizing waste overall also requires that reactors are non-fouling while delivering products with all target functional properties (e.g., gas sorption capacity, conductivity) in high space time yield (STY).

Reactor fouling in wet-chemical nanoparticle synthesis encompasses several mechanisms where material is deposited on reactor surfaces^15^. Flow reactors are likely to foul when solid particles formed in solution adhere to reactor walls resulting in local accumulation (i.e., fouling) and potentially clogging. Both impede the reactor operation, disturb controlled synthetic conditions, and reduce the yield. Reactors that can inhibit fouling by design, e.g., reactors featuring high shear or external forces^16,17^, reactors with low surface to volume ratios^18,19^, jet reactors^20^, two-phase and multiple-phase segmented flow reactors^9,21^, or reactors tuning particle and wall surface chemistry^20,22^, have not seen widespread application in scalable, non-toxic MOF production approaches.

Hydrothermal or solvothermal methods implemented in batch reactors are the most common means for synthesizing large and high-quality MOF crystals in research environments^23,24^. However, these methods have disadvantages of long reaction times, batch-to-batch variations, and generally make use of toxic solvents (e.g., DMF and DEF)^25^. DMF has been restricted for use in chemical synthesis by the European Commission from December 2023^26^. This regulation limits the ability to scale MOFs produced in DMF and accelerates the adoption of MOF syntheses that use alternative green solvents. Water^27^, ethanol^7^, isopropyl alcohol^28^, STEPOSOL MET-10U (*N*,*N*-dimethyl-9-decenamide)^29^ and Cyrene (dihydrolevoglucosenone)^30^ have been reported as green solvents for MOF synthesis. Among these solvents, water has advanced as the most promising for green chemical production of MOFs because of its low toxicity and safety, independence of fossil fuel feedstocks, and limited side-reactions^31–33^. Emerging synthetic approaches, i.e., sono-chemical^34^, microwave^35^, electrochemical^32^, and mechanochemical synthesis^36^, have been reported for faster syntheses of MOFs in batch. However, integration and translation of batch processes to reactors delivering continuous synthetic operation remains a nontrivial objective.

Microfluidic and millifluidic methods have seen particular success offering routes to tighten control over synthetic conditions, continuous operation, and production scale-up for nanomaterials fabrication^37,38^ and biopharmaceutical engineering^39^. Flow reactors have higher mass and heat transfer compared with batch reactors because of their high surface area to volume ratios, fostering higher reaction rates and increased reaction STYs^9,15^. Among these microfluidic and millifluidic approaches, two-phase droplet flow can prevent channel clogging and, by creating a well-defined droplet reactor volume, supports exquisite control of the reaction time (residence time distribution) for enhanced control of particle size compared to single-phase flow designs^40^. In addition, two-phase flow systems provide a promising method for the fast synthesis of MOF particles, as vortices within the droplet flow further increase the mixing and the heat and mass transfer^41^. However, MOF synthesis often remains plagued by slow nucleation rates and many two-phase flow setups require post-synthetic phase separation steps, presenting a persistent challenge for the scale-up of MOF synthesis.

The sono-chemical synthesis technique has proven advantageous for the production of MOFs, attributed to ultrasound-enhanced crystal nucleation rates^34,42^. The prevailing interpretation is that ultrasonic waves induce cavitation as a secondary effect when applied in a liquid medium, creating microbubbles throughout the liquid medium and producing improved MOF crystallization rates compared to conventional heating^34,43–45^. The oscillation and collapse of the cavitation bubbles also serve to break-up agglomerates^46^. Moreover, acoustophoretic effects direct microparticles to the center of reactor, inhibiting sedimentation^47^, thereby preventing clogging. In batch processes, however, uneven control of the reactor temperature can produce poor reproducibility in addition to the extended reaction times and batch-to-batch variations typical of solvothermal approaches. Despite the promise of sono-chemical MOF synthesis, significant aspects of the role of ultrasonic waves (particularly probing the power dependence on MOF synthesis and separating this effect from temperature effects), remain to be explored.

Several MOFs, such as HKUST-1, MOF-5, IRMOF-3, UiO-66, and ZIF-8, have been fabricated in continuous flow reactors with a focus on improving the scalability and green chemical credentials for MOF syntheses^40,48–50^. In more limited cases, a few examples of MOF syntheses in ultrasound-assisted continuous flow reactors in water have been reported^34,51^. The combination of sono-chemical and continuous flow methods has to date, however, been limited to single-phase flow for reaction scale-up^34^ or for modifying the shape of MOFs through ultrasonic etching^51^. Departing from the prevailing single-phase ultrasound-assisted crystallization paradigm for molecular crystals and inorganic materials^34,52^ (and distinct from separated, sequential sono-chemical and slug-flow for amino acid crystallization^53^), we turn our attention here to the prospect of two-phase flow in an ultrasound-assisted reactor platform. This distinct combination specifically serves to minimize clogging problems while determining how ultrasound power promotes STY across varied MOF syntheses. We posit that integrating ultrasound and continuous, two-phase water/gas reactor concepts offers key advantages of both sono-chemical and flow synthesis without additional separations or toxic solvents or carrier phases, whilst also offering well-controlled MOF particle size and enhanced STY for both rapid feedback during process development and, ultimately, scalable production^54,55^. We seek to resolve the apparent tension between stability requirements for two-phase water/gas and ultrasonic wave stimulation, critically offering a route to two-phase flow without liquid carrier phase waste.

**Fig. 1:**
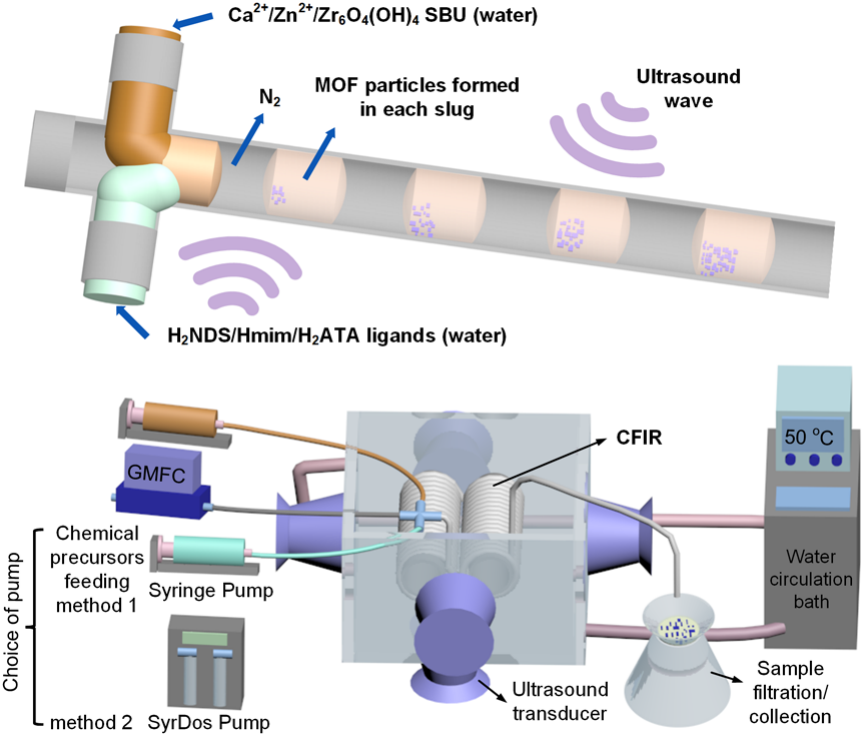
Schematic diagram of the ultrasound-assisted two-phase flow reaction platform. The reaction platform has been developed for the synthesis of sulfonate, imidazolate, and carboxylate MOFs. The reactor is demonstrated for two pump configurations, including continuously operable pumps (SyrDos Pump).

In this work, we first report the continuous, ultrasound-assisted, single-phase flow synthesis of Ca-NDS (water), a Ca-based naphthalenedisulfonate coordination polymer with promising proton conductivity^31^. Then, following on from a parameter-space exploration for Ca-NDS (water) in single-phase flow, we report a second-generation reactor in the form of an ultrasound-assisted, two-phase flow reactor using N_2_ gas as the carrier phase for targeted development as a universal and non-fouling MOF reactor. Figure 1 presents a schematic of this reactor, providing a conceptual overview and highlighting the simultaneous mixing of separately introduced metal and ligand precursor solutions and solvent slug formation as well as the integration of two-phase flow within a coiled flow inverter reactor (CFIR) design with ultrasonic transducers positioned on five sides under dynamic temperature control (circulating water). Crucially, while initial development relied on syringe pumps, we have embedded flexibility in the design and confirm compatibility with fully continuously operable SyrDos pumps as well. We demonstrate this reactor’s capabilities for the aqueous synthesis of Ca-NDS (water) as well as aqueous syntheses of ZIF-8 and UiO-66-NH_2_ MOFs. The reactor, operating at stable temperature, offers a reliable, rapidly transferable reaction platform for diverse MOF chemistries and unambiguously demonstrates the significant role of ultrasound power in increasing STYs.

## Results and discussion

### Ultrasound-assisted single-phase flow synthesis of Ca-NDS (water) in an ultrasonic bath

The Ca-NDS (water) coordination polymer has been reported by Cai et al.^56^, as well as in our previous work^31^ using a solvothermal batch synthesis. However, the large Ca-NDS (water) particles generated by the batch reaction limits their direct use in proton exchange membranes (PEMs), because only small and uniform MOF particles can be used to make homogenous PEMs. Ultrasound-assisted flow synthesis offers a promising route to achieve small and uniform particles in high yield. Firstly, we implemented a synthesis in a single-phase flow reactor placed in an ultrasonic bath to explore the effects of the main parameters of reaction temperature, time, and concentration of precursors.

**Fig. 2:**
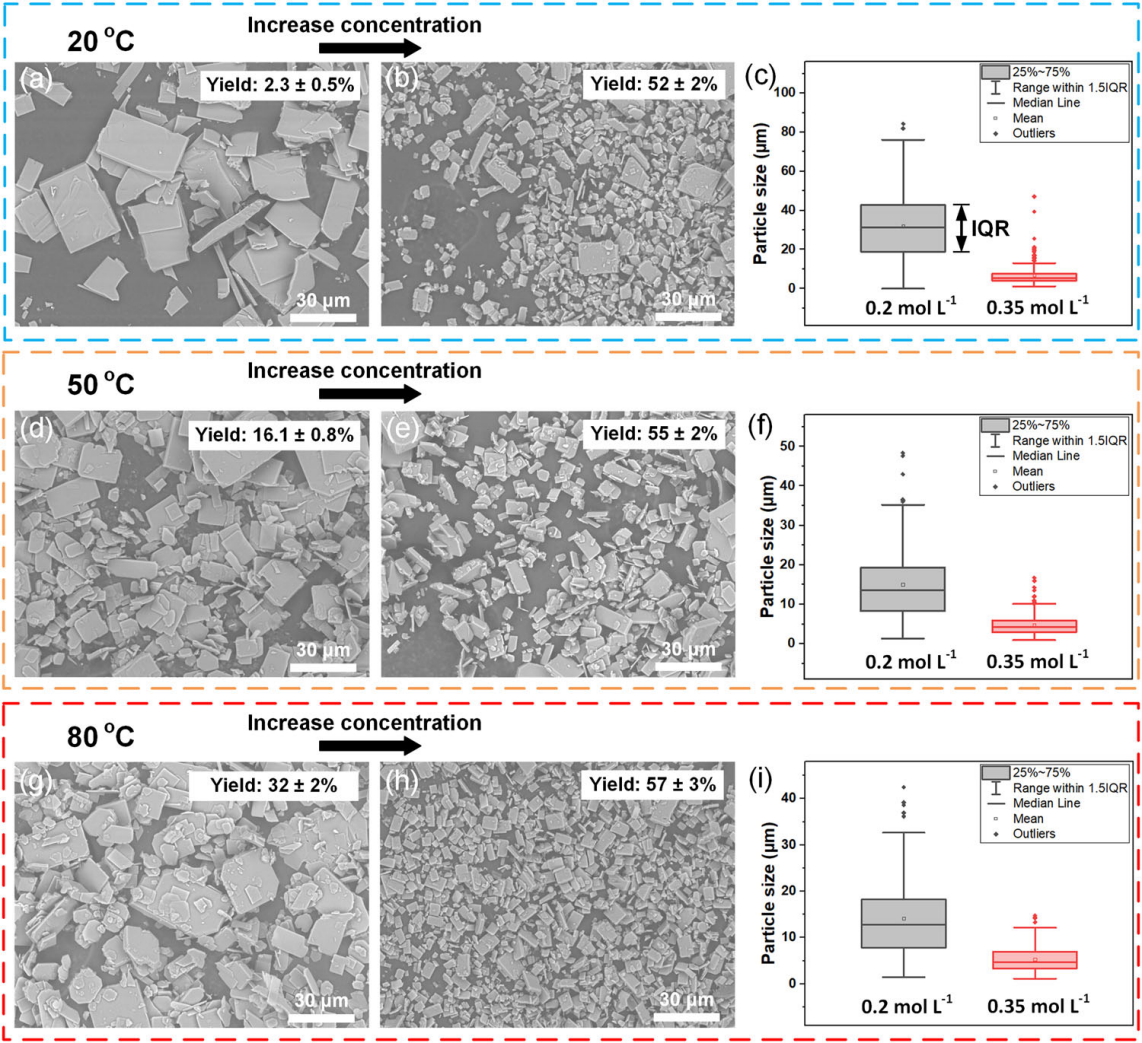
SEM images of Ca-NDS (water) made in single-phase ultrasound-assisted flow. SEM images depict Ca-NDS (water) prepared across (**a**)–(**c**) 20 °C, (**d**)–(**f**) 50 °C, and (**g**)–(**i**) 80 °C. Each row contains images for concentrations of precursor solutions with the same metal-to-ligand ratio at (**a**), (**d**), (**g**) 0.2 M and (**b**), (**e**), (**h**) 0.35 M as well as (**c**), (**f**), (**i**) the corresponding box plots showing the distribution of particle sizes. The horizontal lines in the box plots are the minimum, first quartile, median, third quartile, and maximum. The mean is shown as a square. Outliers (greater than 1.5 times the interquartile range) are shown as additional points. The residence time of these reactions was 1.25 min.

Figure 2 presents the SEM images of Ca-NDS (water) particles made in this reactor. For a reaction temperature fixed at 20 °C, increasing the concentration of precursors from 0.2 to 0.35 M results in a significantly increased reaction yield from 2.3 to 51.8% and a reduction in particle size (median particle size decreased from 31.2 to 5.9 μm, Fig. 2a, b). The distribution of particle sizes also narrowed, with the particle size interquartile range (IQR) reduced from 23.9 to 2.7 μm (Fig. 2c). Similar trends were observed for reaction temperatures fixed at 50 and 80 °C. Comparing the temperature response, yield increased with temperature while particle size and particle size IQR decreased with increasing temperature. While other MOF microfluidic syntheses of other MOFs in organic solvent systems have supported finer particle size and shape uniformity than these single-phase results for Ca-NDS (water)^40,50^ our focus here is on identifying the characteristic features of this sulfonate MOF synthesis to lead onward to optimization in two-phase flow (see below).

The *X*-ray diffraction (XRD) pattern of Ca-NDS (water) synthesized in single-phase flow (Supplementary Fig. 1a) exhibited identical peak positions as for the batch synthesis and as for the pattern calculated from the single-crystal structure. The intensities of the (001) and (003) reflections were enhanced for Ca-NDS (water) synthesized in flow, consistent with preferred orientation in the fine powder sample comprised of plates with top and bottom {001} surface terminations, a habit observed previously in batch-synthesized crystals^31^. Compared with batch-synthesized Ca-NDS (water) particles, the ultrasound-assisted flow synthesis produced much smaller and more uniform particles (Supplementary Fig. 1b, c).

**Fig. 3:**
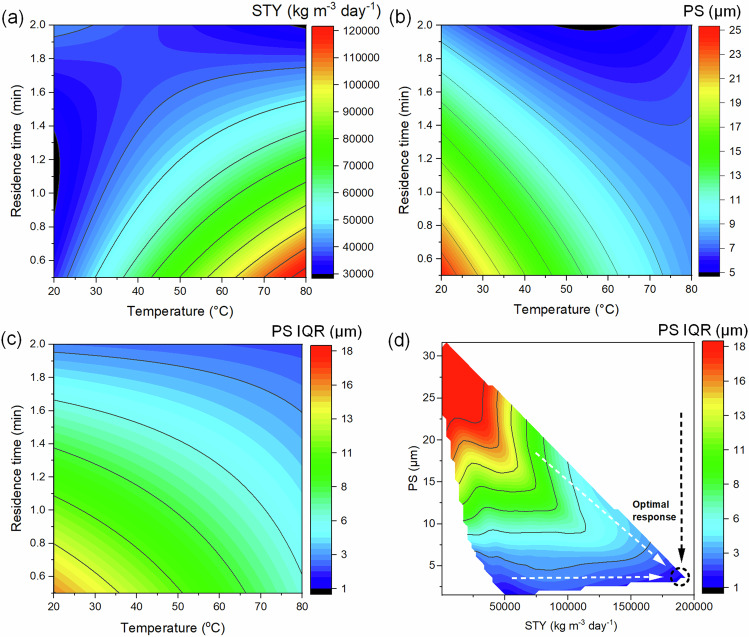
Linear response surface modeling results based on a factorial design of experiments. The response surface models are depicted as contour plots of (**a**) STY, (**b**) PS, and (**c**) PS IQR as a function of synthesis temperature and time at a fixed concentration of reagents (0.275 mol L^−1^). **d** Contour plot depicting the simultaneous optimization of maximum STY and minimum PS and PS IQR. Abbreviations in this figure: Space time yield (STY), Particle size (PS), Particle size interquartile range (PS IQR).

To probe the interactions between key parameters (reaction temperature, time, and concentration of precursors), we employed a factorial experimental design. Specifically, we implemented a Box-Behnken experimental design comprising 12 different experiments varying each parameter and three repeats at intermediate values (15 total experiments). Supplementary Fig. 2a shows a visualization of this design. All reaction parameters from the Box-Behnken design and the corresponding reaction results are listed in Supplementary Table 1. The standard deviations for yield, STY, particle size, and particle size IQR in these three repeated experiments were 1.2%, 1.4 × 10^3^ kg m^−3^ day^−1^, 0.4 and 0.4 μm, respectively. These results were in turn used for linear response surface modeling to document the trade-offs between reaction parameters and STY, particle size, and particle size IQR (Supplementary Note 1, Supplementary Tables 2–6, Fig. 3 and Supplementary Fig. 2).

Figure 3a–c present contour plots depicting the response surface modeling as a function of temperature and residence time at a fixed reagent concentration of 0.275 mol L^−1^. Supplementary Fig. 2b–d presents additional contour plots as a function of the concentration of reagents and residence time at a fixed temperature (50 °C). These findings highlight that increasing temperature, reducing residence time, and increasing the concentration of reagents maximize the STY (Fig. 3a, Supplementary Fig. 2b). In contrast, the smallest particle sizes and particle size IQRs are achieved at high temperature, long residence times, and high concentrations of reagents (Fig. 3b, c, Supplementary Fig. 2c, d). The apparent trade-off in short residence times for high STY and long residence times for small particle sizes and particle size IQRs, however, can be resolved by the compensating control of particle size and particle size IQR via the concentration of reagents. That is, considering the combinations accessible in the reactor for the simultaneous objectives of maximum STY and minimum particle size and particle size IQR (Fig. 3d), a single optimum condition was determined offering high STYs accompanied by small and uniform particle sizes. This characteristic association indicates conditions dominated by crystal nucleation. In contrast, low STYs coincident with larger particle sizes and particle size IQRs reflect a more crystal growth-dominated evolution of the particulate reaction products. The optimal conditions observed experimentally corresponded to the highest STY of 1.6 × 10^5^ kg m^−3^ day^−1^ with a production rate of 59 g h^−1^ for an intermediate reaction temperature of 50 °C, the shortest reaction time (30 s), and the highest concentration (0.35 M) used in the Box-Behnken design, with a correspondingly small particle size and particle size IQR of 5.1 and 3.6 μm, respectively.

To verify the preservation of functional properties, mixed matrix membranes (MMMs) were prepared for proton conductivity testing. Supplementary Fig. 3a, b shows a homogeneous dispersion of the Ca-NDS (water) crystals in the MMM (denoted Ca-NDS (water)-MMM-1) by light microscopy and cryo-SEM. A cross-section observed by cryo-SEM was used to estimate the thickness of the hydrated membrane at approximately 112 μm. Supplementary Figs. 4, 5 present EDS mapping of the membrane both in cross section and in plan view, confirming the dispersion of Ca-NDS (water) particles throughout the membrane. EIS (Supplementary Fig. 3c) confirmed the proton conductivity of Ca-NDS (water)-MMM-1 as 0.97 ± 0.05 mS cm^−1^ at 80 °C in 95% relative humidity (RH). Using EIS across multiple temperature conditions, an Arrhenius plot (Supplementary Fig. 3d) was used to determine the activation energy, *E*_*a*_, for proton conduction in the membrane as 32 ± 3 kJ mol^−1^ or 0.33 ± 0.03 eV, a value consistent with a predominantly Grotthuss mechanism (*E*_*a*_ < 0.4 eV)^57^.

### Ultrasound-assisted continuous MOF reactor: two-phase flow

We next developed a second-generation setup in order to better control the reaction parameters in ultrasound-assisted flow synthesis and to generalize the reactor to the aqueous synthesis also of ZIF-8 and UiO-66-NH_2_. This setup added a water circulation bath to control the reaction temperature dynamically. The ultrasonic bath used in the initial single-phase flow synthesis showed linear temperature increases with increasing reaction times (Supplementary Fig. 6a) for set temperatures of 20 and 50 °C, consistent with the addition of heat to the bath from the ultrasound exposure. In contrast, the second-generation platform preserved temperature stability to within 0.25 °C (Supplementary Fig. 6b and c). This second-generation reactor also supported changing the ultrasound power to further probe the effect of this key parameter on the aqueous syntheses of Ca-NDS (water), ZIF-8, and UiO-66-NH_2_.

First, we replicated the single-phase flow synthesis of Ca-NDS (water) in the second-generation reactor. Under matched conditions of temperature, time, and concentration of precursors (now variable ultrasound power), the yield and STY of Ca-NDS (water) increased with increasing ultrasonic power (Supplementary Table 7), while particle size and particle size IQR decreased with increasing ultrasonic power (Supplementary Table 8). We attribute these observations to the vigorous microscale mixing and diffusion of the solute and increased nucleation rates under sonication^42^. Although this updated reaction platform was promising, clogging was observed during the single-phase synthesis of Ca-NDS (water) after running the reaction for more than 10 min.

**Fig. 4:**
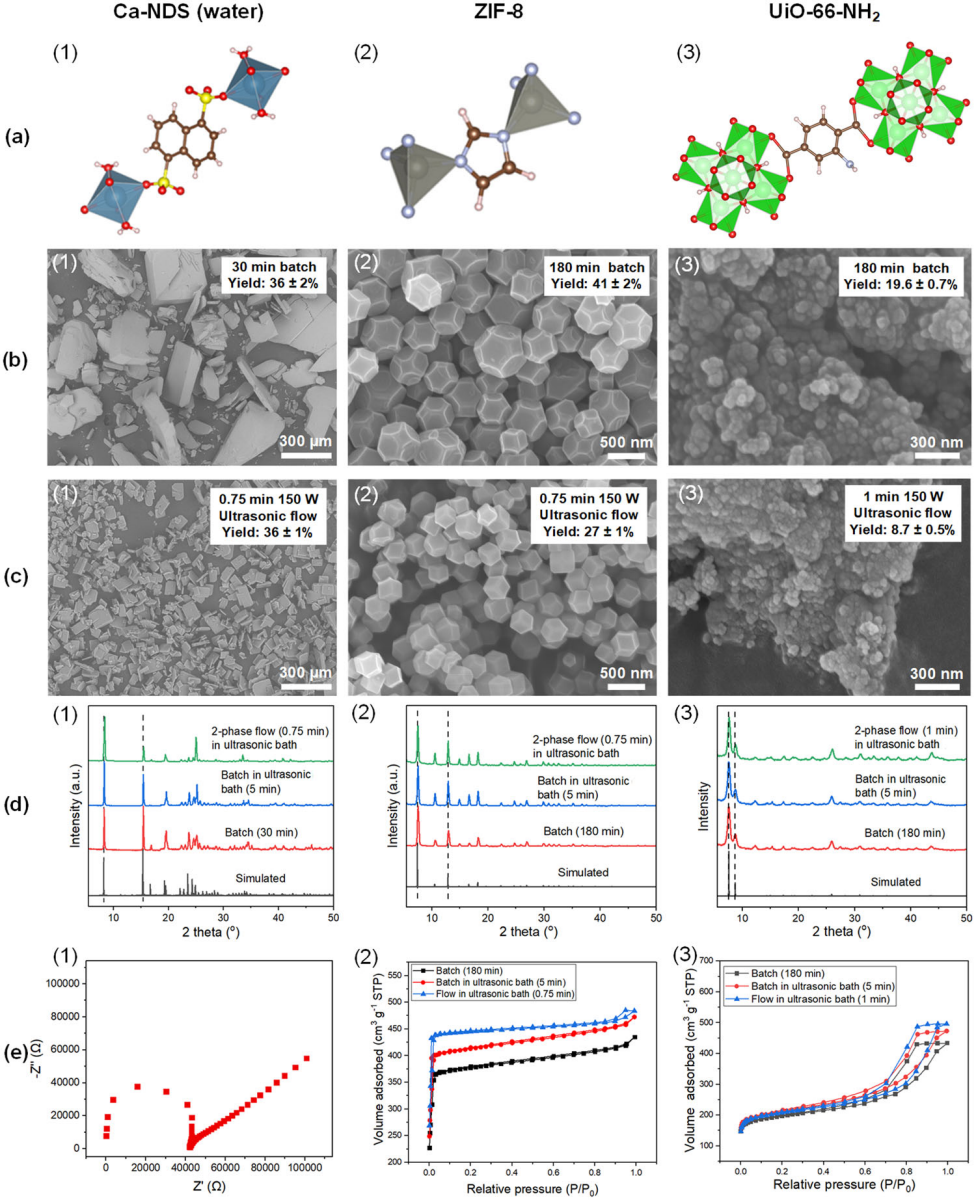
Sulfonate, imidazolate, and carboxylate MOFs prepared in the two-phase ultrasound-assisted flow reactor. **a** Polyhedral representations of Ca-NDS (water), ZIF-8 and UiO-66-NH_2_. **b, c** SEM images of Ca-NDS (water), ZIF-8, and UiO-66-NH_2_ particles made in (**b**) batch and (**c**) ultrasound-assisted two-phase flow. **d** XRD patterns of Ca-NDS (water), ZIF-8, and UiO-66-NH_2_ powders made in batch and ultrasound-assisted two-phase flow compared with simulated patterns. For the simulated patterns, the unit cells were taken from published structures deposited in the Cambridge Crystallographic Data Center (CCDC) for Ca-NDS (water)^56^ (CCDC 152303), ZIF-8^80^ (CCDC 864312), and UiO-66-NH_2_^81^ (CCDC: 1405751). **e** EIS Nyquist plot of Ca-NDS (water)-MMM-2 measured at 80 °C and 95% RH, and N_2_ adsorption-desorption isotherms of ZIF-8 and UiO-66-NH_2_ made in batch and in ultrasound-assisted two-phase flow. The isotherms plotted as black squares for the batch syntheses without ultrasound, red circles for the batch syntheses in an ultrasonic bath, and blue triangles for the two-phase ultrasound-assisted flow syntheses.

In order to overcome this limitation arising from clogging and thereby achieve a more than ten-fold increase in the continuous operation time (albeit with a five-fold reduction in production rate, see below), we further added N_2_ gas as the second phase for ultrasound-assisted two-phase flow synthesis. In addition to Ca-NDS (water), we also demonstrate this two-phase flow reactor for the synthesis of ZIF-8 and UiO-66-NH_2_ in aqueous solvent. These MOFs were selected to encompass diverse functional group chemistries, from the sulfonate linkers of Ca-NDS (water) to the imidazolate linkers in ZIF-8 and carboxylate linkers in UiO-66-NH_2_ (Fig. 4a). To demonstrate the facile transfer of batch conditions to the ultrasound-assisted continuous reactor platform, we have selected these MOFs based on detailed reports of their synthesis in aqueous conditions^27,58^. Figure 4b, c present the corresponding SEM images for these three MOFs, comparing the products prepared in batch and in ultrasound-assisted two-phase flow. Compared with the batch samples, smaller and more uniform particles were obtained from the two-phase flow for all three materials. The Ca-NDS (water) particles are consistently rectangular plates, with some appearing edge-on in SEM imaging, and ZIF-8 are well-faceted truncated cuboctahedra of consistent size.

Batch and two-phase flow syntheses produced products with the same peak positions in XRD as expected from unit cell calculations (Fig. 4d), confirming that ultrasound did not alter the crystal structures produced. The functional group chemistry of each material synthesized in the ultrasound-assisted two-phase flow reactor (Ca-NDS (water), ZIF-8, and UiO-66-NH_2_ MOFs) was further confirmed by ATR-FTIR (Supplementary Fig. 7), and the thermal response characteristics were examined by thermogravimetric analyses (TGA) (Supplementary Fig. 8). SEM-EDS and *X*-ray photoelectron spectroscopy (XPS) analyses were used to confirm the elemental composition, homogeneity, and chemical states relative to the reported unit cell structures of Ca-NDS (water) (Supplementary Figs. 9 and 10a–c, Supplementary Tables 9, 10), ZIF-8 (Supplementary Figs 11 and 10d–f, Supplementary Tables 11, 12), and UiO-66-NH_2_ (Supplementary Figs 12 and 10g–i, Supplementary Tables 13, 14). Together, this structural and chemical characterization, outlining a suitable set of laboratory-scale characterization techniques for rapid assessment of reactor performance matched to the objective of demonstrating reactor transferability across MOF chemistries, confirmed the reliable synthesis of the target materials.

Each of the materials also showed matched or superior functional performance properties compared to the samples prepared using batch syntheses. A membrane prepared using Ca-NDS (water) prepared in two-phase flow denoted Ca-NDS (water)-MMM-2 (Fig. 4e-1, thickness measured by cryo-SEM in Supplementary Fig. 13a), exhibited a proton conductivity from EIS of 0.94 ± 0.03 mS cm^−1^ at 80 °C in 95% RH. Corresponding pellets exhibited a proton conductivity of 1.48 ± 0.05 mS cm^−1^ at 80 °C in 95% RH (Supplementary Fig. 13b and Supplementary Table 15). These proton conductivity results of Ca-NDS (water) made in two-phase flow were slightly higher than those reported in our previous work^31^. We attribute this improvement to the smaller and more uniform Ca-NDS (water) particles made in the two-phase flow which assist in the preparation of homogenous membranes and pellets with reduced void space.

For the MOFs ZIF-8 and UiO-66-NH_2_, we assessed their properties by N_2_ adsorption-desorption isotherms. ZIF-8 isotherms presented a rapid increase at low relative pressure (P/P_0_ < 0.1) and typical type-I isotherm behavior^59^ (Fig. 4e-2), consistent with the microporous structure of ZIF-8. ZIF-8 synthesized in the second-generation ultrasound-assisted two-phase flow reactor exhibited a surface area of 1886 m^2^ g^−1^ and a pore volume of 0.721 cm^3^ g^−1^. The specific surface area is very close to the theoretical maximum of 1947 m^2^ g^−1^^60^. Both surface area and pore volume values were higher than for the corresponding batch-synthesized samples (Supplementary Table 16), consistent with the formation of high quality ZIF-8 crystals with significant accessible pore space^60^. The N_2_ isotherms recorded on UiO-66-NH_2_ samples were classified as type IV isotherms with a clear hysteresis loop^61^ (Fig. 4e-3). UiO-66-NH_2_ synthesized in two-phase flow exhibited a surface area of 787 m^2^ g^−1^ and a pore volume of 0.756 cm^3^ g^−1^. These values were similar to those recorded on samples made in an ultrasonic bath but were higher than batch synthesized samples without ultrasound (Supplementary Table 17). Together, the EIS and N_2_ isotherms indicated that the Ca-NDS (water), ZIF-8, and UiO-66-NH_2_ crystals prepared by ultrasound-assisted two-phase flow exhibit equal or superior quality to matched aqueous-phase batch synthesis products.

**Fig. 5:**
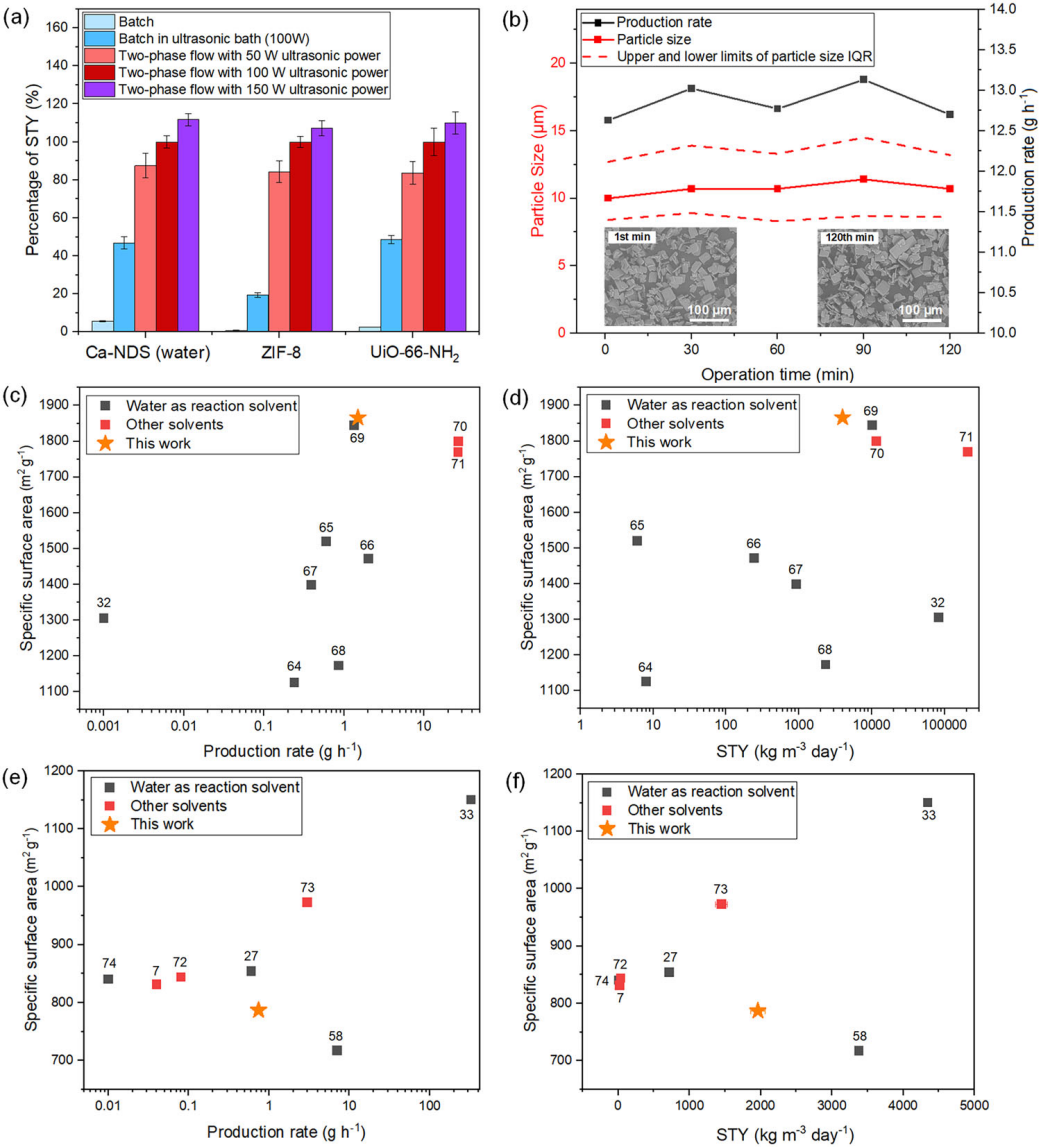
Continuous, ultrasound-assisted production of MOFs. **a** Normalized STYs of MOFs made in batch and two-phase flow (100% STY is taken as the STY in two-phase flow with 100 W ultrasonic power). **b** Production rate and particle size for continuous operation of the ultrasound-assisted two-phase flow reactor, showing sustained production of Ca-NDS (water) across 120 min with a 0.75 min residence time and 150 W ultrasonic power at 50 °C. Specific surface area of ZIF-8 made in the two-phase flow (this work, yellow star) as a function of (**c**) production rate and (**d**) STY in comparison with previously reported syntheses. **e, f** Specific surface area of UiO-66-NH_2_ made in the ultrasound-assisted two-phase flow (this work, yellow star) as a function of (**e**) production rate and (**f**) STY in comparison with previously reported syntheses. Previous results are shown as black squares where water was used as the solvent and red squares where other solvents were used. Note: Error bars in Fig. 5c–f are smaller than the size of the symbols plotted. Error estimates (including where available in previous reports) are included in Supplementary Tables 21, 22.

Our reactor design specifically targets the flexible adjustment of ultrasound power between 50 and 150 W allowing the study of this parameter across all three materials Ca-NDS (water), ZIF-8, and UiO-66-NH_2_. Figure 5a depicts the dependence of STY on ultrasound power, normalized to the STY at 100 W for consistent comparison independent of differences in overall reactivity of the three syntheses at the available flow rates. We include batch conditions as well as batch conditions in an ultrasonic bath as key benchmarks. Control experiments of the continuous reactor platform without ultrasound were attempted but were not feasible due to clogging, attributed to the growth of larger crystals than in the ultrasound-assisted case. These STYs were calculated from the total weight of the collected products, justified by the phase purity and the retained or improved properties (Fig. 4). The STY and production rate results are also summarized in Supplementary Tables 18–20. We note that the STYs in two-phase flow are calculated using the total reactor volume; the yields per volume of solvent used per unit time are approximately double the reported STYs. We observed a consistent trend of increasing STY with increasing ultrasound power across all three linker functional group families. With increasing ultrasound power, smaller particles of Ca-NDS (water) and ZIF-8 were also produced (Supplementary Figs 14, 15), further supporting the enhanced nucleation effects of the ultrasound power. As UiO-66-NH_2_ particles were too aggregated to distinguish easily primary particle size, we have not included particle size result for UiO-66-NH_2_ (Supplementary Fig. 16). Further advances in future to incorporate online monitoring may further elaborate the details of the critical mechanisms outlined by these results regarding the effect of ultrasound power on STY at the scale of the powder sample.

In this work, our primary focus is on the prospects for scalable, non-fouling MOF production combining sono-chemical and continuous flow approaches. The replacement of toxic solvents is one of the twelve principles of green chemistry^62^, and the one that is embedded within this aqueous reactor with gas as the carrier phase. Nevertheless, waste considerations as well as energy consumption, among many other factors, also govern whether a process offers a green chemical advance. We have calculated *E* factors (defined as the mass ratio of waste to product)^62^ for the reactions reported here (Supplementary Tables 18–20). The *E* factor for Ca-NDS (water) in two-phase flow and using an ultrasound power of 150 W with a reaction time of 0.75 min matches the batch reaction time of 30 min, both with a value of 18.73 (with water) or 1.78 (excluding water). These values track the yields directly, but the two-phase reactor platform offers an order of magnitude higher STY. This fixed relationship to yield is reproduced in the *E* factors for ZIF-8 and UiO-66-NH_2_. For ZIF-8 in two-phase flow and using an ultrasound power of 150 W with a reaction time of 0.75 min, the *E* factor is 360 (42 without water) compared to an *E* factor of 240 (28 without water) in a batch reaction running for 180 min. For UiO-66-NH_2_ in two-phase flow and using an ultrasound power of 150 W with a reaction time of 1 min (highest STY), the *E* factor is 390 (74 without water) compared to an E factor of 170 (32 without water) in a batch reaction running for 180 min. The STYs are in these cases two orders of magnitude greater in two-phase flow than in batch. These findings indicate a consistent trade-off in STY and *E* factor (following the yield) in this reactor. We have also considered solvent intensity (defined as the mass ratio of all solvent used excluding water to product)^63^, which describes the Ca-NDS (water) and ZIF-8 reactions with a metric of zero. For UiO-66-NH_2_ two-phase flow conditions giving the highest STY, the solvent intensity value is 52 compared to a value of 23 for the 180 min batch conditions based on the use of glacial acetic acid in this reaction (all lower than in a comparable DMF-based synthesis). We highlight that no substantial re-optimization was carried out for the ultrasound-assisted two-phase flow syntheses of either ZIF-8 or UiO-66-NH_2_. Further optimization of reaction parameters to improve yield and STY while also recycling chemical precursors to maximize reagent utilization and minimize environmental pollution offer routes for future progress in waste reduction for this type of reactor. Lifecycle assessment to also consider energy consumption would then also offer a more complete picture of the green chemistry impact, especially when compared to our focus on non-toxic, scalable synthesis.

Finally, to highlight this reactor’s capabilities for a fully continuous mode of operation, the syringe pumps were further replaced by two SyrDos pumps to continuously feed precursors (total flow rate of 6 mL min^−1^, flow rate of N_2_ gas was 6 mL min^−1^). Returning to the ultrasound-assisted two-phase flow continuous synthesis of Ca-NDS (water) with 0.75 min residence time at 50 °C under 150 W ultrasonic power, we operated the reactor for more than 120 min without interruption. As shown in Fig. 5b, this reaction platform demonstrated a sustained production rate of 12.9 ± 0.4 g h^−1^ in the synthesis of Ca-NDS (water) with no reduction in the production rate or change in particle size observed during this extended continuous operation. Moreover, we observed no evidence of reactor fouling. The reaction yield remained constant at 35.8 ± 0.5% and the STY was consistently 3.43 × 10^4^ ± 5 × 10^2^ kg m^−3^ day^−1^, exhibiting smaller deviations than the errors estimated from three separate flow syntheses using syringe pumps under otherwise identical reaction conditions (Supplementary Fig. 17 and Supplementary Table 18). Not only does the reactor platform offer rapid transferability across different aqueous-phase synthesis, but this evidence on continuous operation additionally highlights the suitability of this reaction platform design for scalable production.

The highest STYs (using syringe pumps) for Ca-NDS (water), ZIF-8, and UiO-66-NH_2_ MOFs were 3.4 × 10^4^ ± 1 × 10^3^, 4.0 × 10^3^ ± 2 × 10^2^, and 2.0 × 10^3^ ± 1 × 10^2^ kg m^−3^ day^−1^, respectively. To contextualize these results, we have drawn together other previously reported aqueous-phase syntheses of ZIF-8^32,64–71^ and UiO-66-NH_2_^7,27,33,58,72–74^ that report the necessary details for comparison of production rate, STY, and specific surface area (Fig. 5c–f and Supplementary Tables 21, 22). Only two previous reports, including one of our own, have reported the synthesis of Ca-NDS (water) with previous work focused on the structure rather than production rate. Nevertheless, STYs of the order of 10^3^−10^5^ kg m^−3^ day^−1^ and production rates of the order 10^1^ g h^−1^ have been considered of scalable and commercial interest in other systems^75,76^. Our ZIF-8 synthesis stands out as delivering specific surface areas and production rates among the highest reported to date, excluding approaches with different solvents or additives (Fig. 5c and d), whereas our UiO-66-NH_2_ synthesis is within the spread of previous reports (Fig. 5e and f). The surface area production rate (SAPR) has also been put forward as an important figure of merit^77,78^. At 7.5 × 10^9^ m^2^ m^−3^ day^−1^ for ZIF-8 and 1.6 × 10^9^ m^2^ m^−3^ day^−1^ for UiO-66-NH_2_, our reactor offers competitive SAPR values for both ZIF-8 and UiO-66-NH_2_ (maximum prior values of 10^6^−10^11^ m^2^ m^−3^ day^−1^ for ZIF-8 and 10^6^−10^9^ m^2^ m^−3^ day^−1^ for UiO-66-NH_2_, Supplementary Tables 21, 22).

While several of the previously reported STYs, production rates, and SAPRs are higher than those we report here, we would highlight that the strength of our approach is primarily its rapid transferability to other systems, i.e., the generalization of the reactor platform. No substantial re-optimization was carried out for the syntheses of either ZIF-8 or UiO-66-NH_2_, reflecting that in many cases competitive figures of merit, improved relative to batch conditions, are rapidly achievable using the ultrasound-assisted two-phase continuous reactor platform. We also note that our reactor is a small laboratory scale flow reactor with 9 mL volume in our reaction platform, and so the competitive production of high-quality crystals and generalization across distinct functional group chemistries highlights the advantages for larger or parallelized reactors of ultrasound-assisted aqueous synthesis in two-phase flow. Using N_2_ as the carrier phase, moreover, ensures facile separation and reduced waste (offering an oil- and organic solvent-free two-phase reactor solution).

In summary, we report an ultrasound-assisted two-phase flow reactor for the aqueous-phase continuous production of the sulfonate coordination polymer Ca-NDS (water), as well the imidazolate MOF ZIF-8 and the carboxylate MOF UiO-66-NH_2_. By using N_2_ gas as the carrier phase, this reactor eliminates steps for the chemical or mechanical separation of a liquid carrier phase. The reactor supports adjustable ultrasound power, enabling the isolation of the effects of this parameter. Ultrasound power consistently increases yield and reduces particle size and the width of the particle size distribution, outlining a consistent enhancement in the rate of nucleation. The reactor nevertheless produces highly faceted Ca-NDS (water) and ZIF-8 crystals with high quality proton conductivities (1.48 ± 0.05 mS cm^−1^ in pellet form and 0.94 ± 0.03 mS cm^−1^ in MMM form at 80 °C in 95% RH) and gas sorption (1886 m^2^ g^−1^), respectively. Using the ultrasound-assisted two-phase flow reactor, the reaction yields at 50 °C were 36 ± 1, 27 ± 1 and 8.7 ± 0.5% for Ca-NDS (water), ZIF-8, and UiO-66-NH_2_, respectively, and we recorded STYs of 3.4 × 10^4^ ± 1 × 10^3^, 4.0 × 10^3^ ± 2 × 10^2^, and 2.0 × 10^3^ ± 1 × 10^2^ kg m^−3^ day^−1^ for Ca-NDS (water), ZIF-8, and UiO-66-NH_2_, respectively. Moreover, we have demonstrated that the reactor can be operated with continuous precursor feeds and without fouling for more than 2 h with a sustained production rate and crystal product quality. Together, these results demonstrate the generalizable and scalable green chemical synthesis of coordination polymers and MOFs using ultrasound-assisted continuously operable reactors. The ultrasound-assisted design may, in turn, boost production rates further in application to reactors with scaled up volumes for efficient MOF synthesis with reduced environmental impact.

## Methods

### Materials

1,5-Naphthalenedisulfonic acid tetrahydrate (H_2_NDS, Molecular Weight, M_W_, 360.36 g mol^−1^), methanol (≥99.9%), polyvinylpyrrolidone (PVP, M_W_ 360,000 g mol^−1^), and polyvinylidene fluoride (PVDF) (PVDF, M_W_ 534,000 g mol^−1^) were purchased from Sigma Aldrich (Merck Group). Calcium nitrate hemi(pentahydrate) (M_W_ 236.15 g mol^−1^), 2-aminoterephthalic acid (99%, H_2_ATA, M_W_ 181.15 g mol^−1^), zirconyl chloride octahydrate (98%, M_W_ 322.25 g mol^−1^) were purchased from Thermo Fisher Scientific. Zinc acetate dihydrate (M_W_ 219.5 g mol^−1^) and 2-methylimidazole (99%, Hmim, M_W_ 82.11 g mol^−1^) were ordered from Fluorochem. Sodium hydroxide (NaOH, M_W_ 40 g mol^−1^) and glacial acetic acid were purchased from Fisher Chemical.

### Batch synthesis

#### Synthesis of Ca-NDS (water)

The synthetic procedure was similar to our previously reported study^31^. Briefly, calcium nitrate (165 mg, 0.7 mmol) was dissolved in deionised (DI) water (between 1 and 1.7 mL) as solution 1. H_2_NDS (252 mg, 0.7 mmol) was dissolved in DI water (between 1 and 1.7 mL) as solution 2. Next, for solvothermal synthesis, solutions 1 and 2 were mixed in a 20 mL glass vial and heated in a dry bath heater (Thermo Fisher Scientific) at 50 °C for 30 min. Alternately, for sono-chemical batch synthesis, the 20 mL glass vial containing the mixed solution was immersed in an ultrasonic bath (GT SONIC-D3, 40 kHz, 100 W) at a temperature of 50 °C for 2 min. After the reaction, white solids were recovered by vacuum filtration and oven dried overnight at 80 °C.

#### Synthesis of ZIF-8

The synthesis process followed reported procedures^64^. Zinc acetate dihydrate (286 mg, 1.3 mmol) was dissolved in DI water (15 mL) as solution 1. Hmim (3736 mg, 45.5 mmol) was dissolved in DI water (15 mL) as solution 2. Next, for solvothermal synthesis, solutions 1 and 2 were mixed in a 40 mL glass vial and heated in a dry bath heater at 50 °C for 180 min. Alternately, for sono-chemical synthesis, the 40 mL glass vial containing the mixed solution was immersed in an ultrasonic bath at a temperature of 50 °C for 5 min. After the reaction, white precipitates were separated via centrifugation at 6800 rpm for 2 min. Collected samples were soaked in methanol for two days before drying overnight in an oven at 80 °C.

#### Synthesis of UiO-66-NH_2_

The synthesis process followed previously reported procedures^58^. Zirconyl chloride octahydrate (1288 mg, 4 mmol) was dissolved in DI water (12 mL) and glacial acetic acid (5 mL) as solution 1. This solution was heated at 60 °C for 2 h and allowed to cool down to room temperature before carrying out subsequent reactions. H_2_ATA (724 mg, 4 mmol) and NaOH (320 mg, 8 mmol) were dissolved in DI water (20 mL) as solution 2. Next, for solvothermal synthesis, solutions 1 and 2 were mixed in a 40 mL glass vial and heated in a dry bath heater at 50 °C for 180 min. Alternately, for sono-chemical synthesis, the mixed solution was immersed in an ultrasonic bath at a temperature of 50 °C for 5 min reaction time. After the reaction, yellow precipitates were separated via centrifugation at 6800 rpm for 2 min. Collected samples were soaked in methanol for two days before drying overnight in an oven at 80 °C.

### Ultrasound-assisted continuous single-phase flow synthesis

#### Ultrasound-assisted single-phase flow synthesis of Ca-NDS (water) in an ultrasonic bath

Glass syringes (25 mL, SGE) were used to deliver chemical precursors (chemical solutions are reported in 3.2.1). A T-mixer (P-713, IDEX, modified with a drill to give an internal diameter of 1.6 mm to avoid clogging) was used to mix all precursors in the inlet of CFIR which used 4.5 m tubing (PFA, outer diameter 1/8 inch, inner diameter 1/16 inch, Adtech) coiled around a plastic tube (diameter ~2 cm). The reactor volume was accordingly taken as 9 mL. The CFIR was immersed in an ultrasonic bath (GT SONIC-D3, 40 kHz, ultrasonic power 100 W). The solution 1 and flows were initiated once the water bath reached the set temperature. The reaction temperature was varied between 20 and 80 °C, the residence time (determined by the flow rates) was varied between 0.5 and 2 min, and the concentration of precursors was varied between 0.2 to 0.35 M. Ca-NDS (water) particles were collected directly in the outlet of CFIR reactor through filtration and then dried overnight in an oven at 80 °C. A schematic diagram of this single-phase flow synthesis is shown in Supplementary Fig. 18.

#### Ultrasound-assisted single-phase flow synthesis of Ca-NDS (water) in a dedicated reactor

To better control reaction temperature and explore the effect of ultrasonic power (varied between 50 and 150 W), a dedicated stainless steel reaction vessel (12 × 12 × 7 cm) with an acrylic lid was constructed. Five ultrasonic transducers (Beijing Yongda Ultrasonic Co., Ltd.) were mounted on each of the walls (other than the lid), powered by an ultrasonic generator (BJV 300, Beijing Yongda Ultrasonic Co., Ltd.). The reaction temperature was controlled by a water circulation bath with refrigeration and heating functions (Fisherbrand^TM^ Isotemp^TM^ R20). The same CFIR (3.3.1) was placed inside the ultrasound chamber. The temperature was varied between 20 and 80 °C, the residence time (determined by the flow rates) was varied between 0.5 and 2 min, the concentration of precursors was 0.275 M, and the ultrasonic power was varied between 50 and 150 W.

#### Ultrasound-assisted continuous two-phase flow synthesis

A two-phase flow reactor was constructed from the single-phase setup (3.3.2) using N_2_ gas as the second phase. Schematic diagrams and a digital photo of the ultrasound-assisted two-phase flow setup are shown in Fig. 1 and Supplementary Figs 19, 20, respectively. Chemical precursors (chemical solutions for synthesizing Ca-NDS (water), ZIF-8, and UiO-66-NH_2_ reported in Sections 3.2.1, 3.2.2, and 3.2.3, respectively) were delivered by glass syringes (25 mL, SGE) or a continuous pump (SyrDos^TM^ 2XLP). The flow rate of N_2_ gas was controlled precisely by a gas mass flow controller (GMFC, FG-201AV, Bronkhorst). A cross connector (P-723, IDEX, modified with a drill to give an internal diameter of 1.6 mm to avoid clogging) was used to mix the precursors and form stable slugs in the inlet of the CFIR (the same reactor as used in Section 3.3.1), forming slugs of consistent volume and concentration. The ratio of flow rates of the chemical precursors to N_2_ gas was 1:1 in order to form a stable slug pattern. The residence time for the synthesis of Ca-NDS (water) and ZIF-8 was 0.75 min, using flow rates for each chemical precursor of 3 mL min^−1^ and a N_2_ gas flow rate of 6 mL min^−1^. The residence time for the synthesis of UiO-66-NH_2_ was 1 min, using flow rates for each chemical precursor of 2.25 mL min^−1^ and a N_2_ gas flow rate of 4.5 mL min^−1^. Both solutions were allowed to flow once the temperature in reaction vessel reached 50 °C, more than one reactor volume of solution was allowed to pass through for achieving stable slug flow before the collection of samples for yield calculations. Ca-NDS (water) was collected directly at the CFIR outlet by filtration. For ZIF-8 and UiO-66-NH_2_, solid products and the reaction solution were collected in a centrifuge tube containing excess water (in order to dilute reaction solutions and avoid further crystal growth during sample collection). The suspension was then centrifuged to isolate the solid product. Sample activation and drying processes were carried out following the same procedures as used for batch synthesis.

### Fabrication of pellets and membranes with Ca-NDS (water)

Proton conductivity measurements of the Ca-NDS (water) synthesized in flow were carried out using both pellet and membrane testing following our previous work^31^. Ca-NDS (water) powders made in the two-phase flow reactor were pelletized under a pressure of 5 ton cm^−2^ for 2 min by using a 5 mm pellet die (Specac). For membrane measurements, Ca-NDS (water) powders were used directly to make MMMs without further processing (i.e., no further grinding required). To form membranes, Ca-NDS (water) particles were incorporated into PVP and PVDF at a weight percent of 60% Ca-NDS (water). Typically, PVDF (45 mg) and PVP (135 mg) were first dissolved in DMF (2.7 mL) by stirring at room temperature for 3 h to obtain a homogeneous gel. Next, Ca-NDS (water) particles (270 mg) were added and dispersed evenly in the gel by vigorous stirring for 1 h at room temperature. The prepared gel was then poured onto a high-temperature resistant glass and cast using an adjustable applicator (BGO 209/2, Biuged Laboratory Instruments Co., Ltd) to form a film of 60 μm thickness. The resulting membranes were dried at 70 °C for 1 h in a vacuum oven to remove excess DMF. Finally, the solidified membranes were washed with DI water three times and then dried at room temperature. MMMs made using Ca-NDS (water) synthesized in single-phase flow and two-phase flow were denoted Ca-NDS (water)-MMM-1 and Ca-NDS (water)-MMM-2, respectively.

### Materials characterization

The surface morphologies of the samples were characterized by light microscopy (LM, Olympus, BX51) and scanning electron microscopy (SEM, Hitachi TM-3030Plus, equipped with a backscattered electron detector and operated at 15 kV accelerating voltage). High resolution SEM images were collected using a Hitachi SU8230 SEM equipped with a cold field emission electron source. An Oxford instruments 150 *X*-Max energy dispersive *X*-ray spectroscopy (EDS) detector, in the Hitachi SU8230 SEM, was used to assess the elemental composition of MOF particles. EDS analysis was conducted with an electron beam accelerated to 10 kV, a probe current of 20 nA, a working distance of 15 mm, and a pixel dwell time of 10 μs. Similar conditions were applied for EDS point analysis on the MOF particles to verify their composition in comparison to their empirical formula. Before the EDS point analysis, MOF particles were drop-cast onto carbon tape and coated with 10 nm Pt to avoid sample charging. For EDS point analysis, at least five areas were collected for each particle to assess the statistical significance of the results. Cryo-SEM was used to measure the thickness, surface morphology, and elemental composition of the hydrated membrane using a Tescan AmberX cryo-PFIBSEM, equipped with a Quorum Technologies PP3010 cryo-stage and an Oxford instruments 150 X-Max energy dispersive EDS detector and operated at a 2 keV electron beam energy. Particle sizes of Ca-NDS (water) and ZIF-8 were measured from SEM images using ImageJ: The average of the length taken as the longest dimension of the particle and the width perpendicular to the length was used as a descriptor of particle size. More than 200 particles were measured for each sample.

Powder XRD patterns were acquired using a Bruker D2 diffractometer (Cu K_α_ *λ* = 1.54184 Å, 2*θ* scan range = 5–50°). Fourier transform infrared (FTIR) spectra were recorded using a Bruker Vertex 80 V FTIR spectrometer with a diamond prism attenuated total reflection crystal. Spectra were collected for wavenumbers between 500 and 4000 cm^–1^ and averaged over 32 scans. Thermogravimetric analyses (TGA) were conducted using a Netzsch STA 449F3 instrument with a heating rate of 10 °C min^−1^ from 30 to 900 °C in a nitrogen atmosphere. The first derivative of the thermogravimetry curve was calculated numerically to extract the temperatures at which there was the steepest change in mass loss. XPS was employed to characterize the surface chemistry of the synthesized MOFs. MOF particles were pressed onto carbon tape and adhered to a standard omicron plate. The experiment was conducted in UHV (<1 × 10^−9^ mbar) on a Specs FlexMod system. The illuminating *X*-ray source was a monochromatic Al K_α_ (*hν* = 1486.7 eV) anode at a power of 400 W and 15 kV. A Specs Phoibos 150 hemispherical analyzer with 1D delay line detectors was used to detect the photoelectrons. The powders were insulating, and so, to reduce any differential charging at the surface which could distort the spectra, an electron flood gun (energy 4 kV and current 75 µA) was used to charge neutralize the sample. Survey spectra were obtained with a pass energy of 50 eV, a step width of 1 eV and a dwell time of 0.1 s. High resolution spectra were collected with a pass energy of 30 eV, a step width of 0.1 eV and a dwell time of 0.2 s. After collecting the spectra, the data were analyzed using CasaXPS software. Binding energies were calibrated using the C-C C1s peak at 284.8 eV and the intensity was calibrated using a previously calculated transmission function for the specific instrument settings. All spectra were fitted with a Shirley background and the peak areas were used to quantify the relative atomic percentages using the respective relative sensitivity functions for each peak. N_2_ adsorption-desorption isotherms were measured using a Nova 800 (Anton Paar) BET surface area analyzer. Prior to measurements, ZIF-8 was degassed for 3 h at 300 °C and UiO-66-NH_2_ was degassed for 5 h at 250 °C. The specific area was determined using the BETSI method^79^. The total pore volume was determined using the adsorption branch of the N_2_ isotherm at P/P_0_ = 0.99 from Nova Anton Paar Kaomi software.

### Proton conductivity testing

Electrochemical impedance spectroscopy (EIS) was carried out on Ca-NDS (water) pellets and MMMs as reported previously^9^. A temperature-controlled humidity chamber (Memmert HCP150) was used for testing between 50 to 80 °C with 95% RH. The proton conductivity (*σ*, mS cm^−1^) was calculated according to:

1 $$\sigma =\frac{L}{{AR}}$$

where *L* represents the thickness of the pellet or membrane sample (cm), *A* refers to the cross-sectional area of the tested sample (cm^−2^), and *R* is the resistance of the sample (*Ω*). *R* was measured by EIS in a two-electrode configuration between frequencies of 100 Hz and 1 MHz using a Gamry 1010E electrochemical workstation.

## Supplementary information

Transparent peer review file

Supplementary information

## Data Availability

The data associated with this paper are openly available from the University of Leeds Data Repository at 10.5518/1609.
