# Supplementary material for: Ultrasound-assisted continuous aqueous synthesis of sulfonate, imidazolate, and carboxylate MOFs with high space time yield
Source: Commun Chem. 2025 May 16;8:154. doi: 10.1038/s42004-025-01548-5 (PMC12084381; doi:10.1038/s42004-025-01548-5)
Supplement: Supplementary file 2 — Supplementary information [file 42004_2025_1548_MOESM2_ESM.pdf]

Supplementary Information

Ultrasound-assisted continuous aqueous synthesis of sulfonate, imidazolate, and carboxylate MOFs with high space time yield

Chao Sun,[a,b] Sang T. Pham,[a,b,c] Sarah L. Boyall,[b] Ben Douglas,[a] Andrew J. Britton,[a,c] Stuart Micklethwaite,[a,c] Thomas W. Chamberlain,[b] Maximilian O. Besenhard,[d] Rik Drummond-Brydson,[a,c] Ke-Jun Wu,[a,e*] Sean M. Collins,[a,b,c*]

a. School of Chemical and Process Engineering, University of Leeds, Leeds LS2 9JT, UK
b. School of Chemistry, University of Leeds, Leeds LS2 9JT, UK
c. Bragg Centre for Materials Research, University of Leeds, Leeds LS2 9JT, UK
d. Department of Chemical Engineering, University College London, London WC1E 7JE, UK
e. Zhejiang Provincial Key Laboratory of Advanced Chemical Engineering Manufacture Technology, College of Chemical and Biological Engineering, Zhejiang University, Hangzhou, 310027, China

*Email: k.wu@zju.edu.cn, S.M.Collins@leeds.ac.uk

Contents:

Supplementary Figure 1 2
Supplementary Note 1 2
Supplementary Figure 2 3
Supplementary Table 1 – Supplementary Table 6 4
Supplementary Figure 3 – Supplementary Figure 6 6
Supplementary Table 7 – Supplementary Table 8 9
Supplementary Figure 7 – Supplementary Figure 10 10
Supplementary Table 9 – Supplementary Table 10 14
Supplementary Figure 11 14
Supplementary Table 11 – Supplementary Table 12 15
Supplementary Figure 12 15
Supplementary Table 13 – Supplementary Table 14 16
Supplementary Figure 13 16
Supplementary Table 15 – Supplementary Table 20 17
Supplementary Figure 14 – Supplementary Figure 16 20
Supplementary Table 21 – Supplementary Table 22 23
Supplementary Figure 17 – Supplementary Figure 20 25
Supplementary References 27

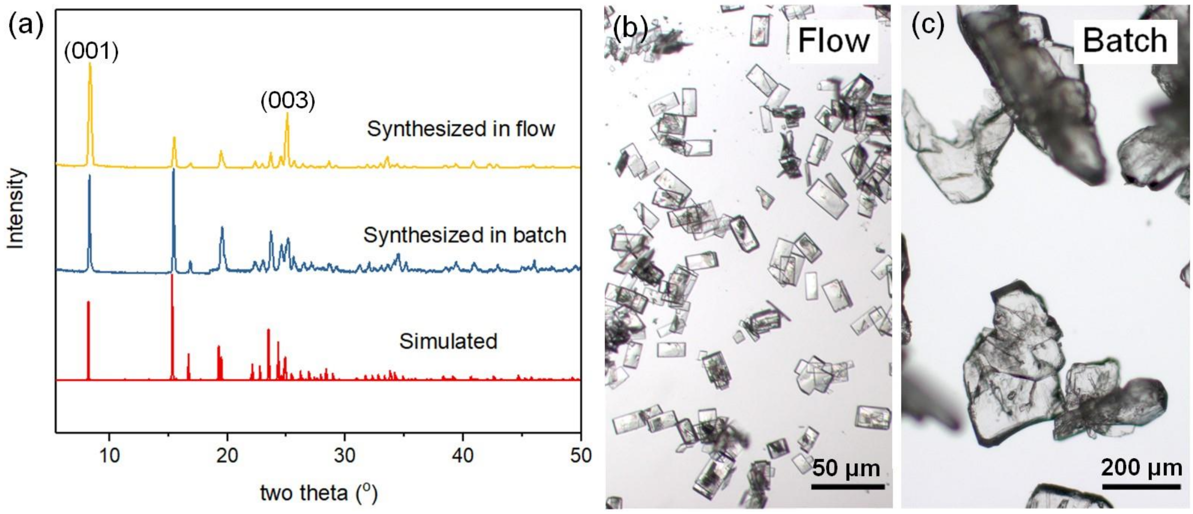

Supplementary Figure 1. (a) Powder XRD patterns of Ca-NDS (water) synthesized in flow and batch alongside a simulated pattern from the reported unit cell [1]. (b)-(c) Light micrographs of Ca-NDS (water) made in (b) flow and (c) batch reactions.

Supplementary Note 1: Linear response surface modelling

Linear response modelling was conducted using Minitab (version 21.3). An analysis of variance was carried out to identify statistically significant terms for linear response model fitting. Terms associated with low probability values (p-values) were excluded from model fitting. The p-values, together with an inspection of the distribution of contributions of all terms, were used to select retained terms, targeting the minimum number of terms to describe the response. Linear response surface modelling was carried out for STY, particle size, and particle size IQR. Equations were used to fit the results across the parameter space: 20 °C ≤ Temperature (T) ≤ 80 °C, 0.5 min ≤ Residence time (t) ≤ 2 min, and 0.2 mol/L ≤ Reagent Concentrations (C) ≤ 0.35 mol L^{-1}.

The values of R^2, the predicted R^2 (R^2 (pred), a calculation involving the removal of each experimental measurement and estimating the quality of the model's prediction of the removed measurements), and the root mean squared deviation (RMSD) for both the Box-Behnken experimental design points and additional verification points (see Supplementary Figure 2a) were considered in selecting the optimized regression equations for STY, particle size, and particle size IQR. The optimized regression equations were chosen based on assessing the relative increase in R^2 for increasing the number of terms, maximizing the predicted R^2 (R^2 (pred)) values, and minimizing the RMSD values. These metrics were balanced with the aim

to minimize the number of terms used in each equation. As summarized in Supplementary Tables 2-5, the optimized regression equations for STY, particle size, and particle size IQR contained 6, 8 and 7 terms, respectively. The coefficients for the corresponding equations are given in Supplementary Table 6. Contour plots for STY and particle size as well as for STY and particle size IQR were then created using these optimized regression equations.

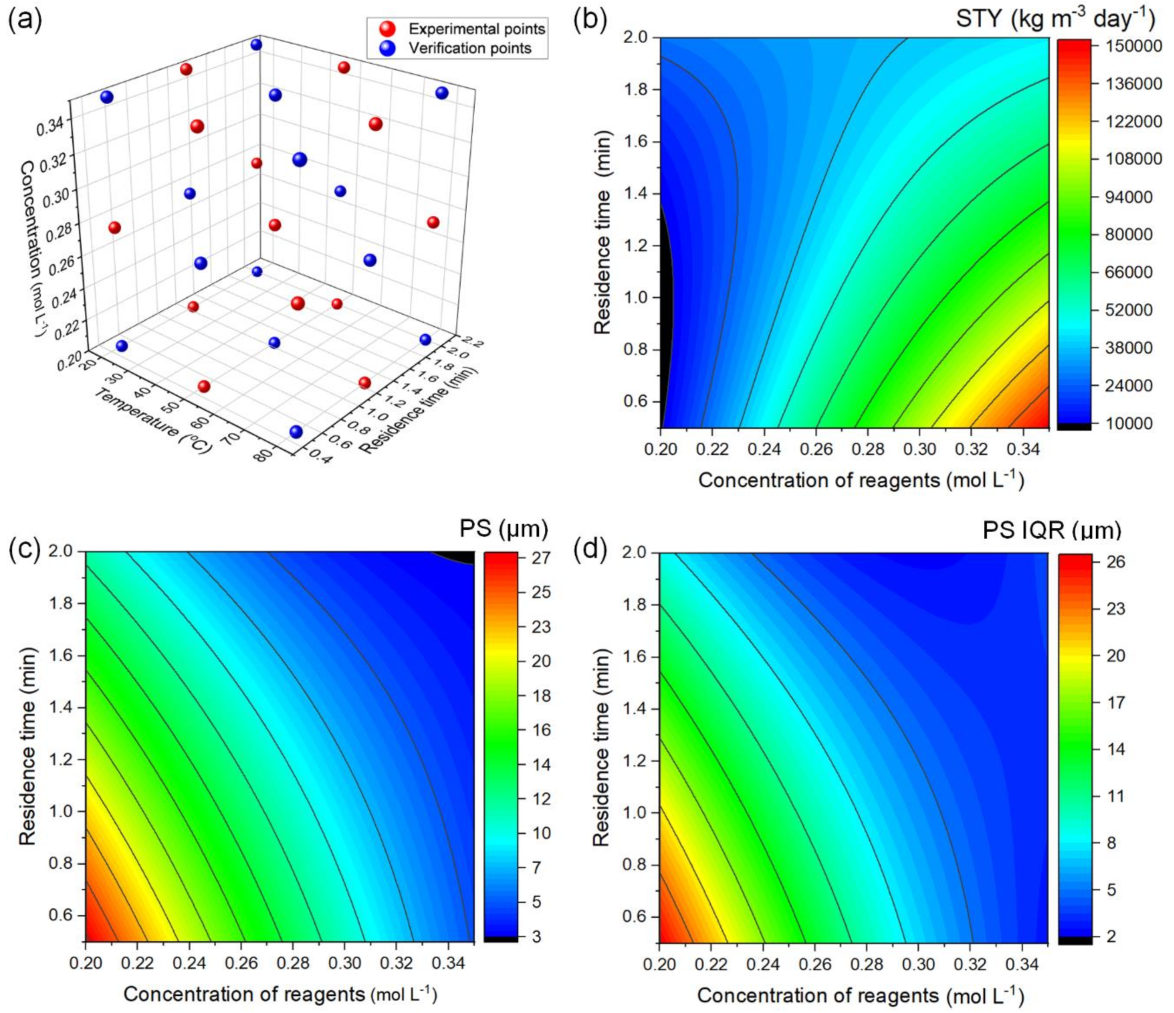

Supplementary Figure 2. (a) Overview of the Box-Behnken factorial design of experiments. The red points mark the conditions used for experimental input for response surface modelling and the blue points mark additional points used for verification of the model. The corresponding coefficients of the response surface modelling are given in Table S6. The response surfaces are represented as contour plots for (b) STY, (c) PS, and (d) PS IQR as a function of the concentration of reagents and the reaction time with the reaction temperature fixed at 50 °C. Abbreviations in this figure: Space time yield (STY), Particle size (PS), Particle size interquartile range (PS IQR).

Supplementary Table 1. Reaction parameters of 15 separate experiments used in the Box-Behnken design and the corresponding reaction results of the ultrasound assisted single-phase synthesis of Ca-NDS (water). Abbreviations in this table: Reaction temperature (T), Residence time (RT), Concentration of reagents (COR), Space time yield (STY), Particle size (PS), Particle size interquartile range (PS IQR).

Experiment No.	T (°C)	RT (min)	COR (mmol mL^{-1})	Yield (%)	STY (kg $m^{-3}day^{-1}$)	PS (μm)	PS IQR (μm)
1	20	0.5	0.275	8.7	24998	25.1	14.9
2	20	2	0.275	48.9	35072	6.9	3.9
3	20	1.25	0.2	2.3	1929	31.2	23.9
4	20	1.25	0.35	46.1	67372	5.9	2.7
5	50	0.5	0.2	0.5	1044	26.1	27.3
6	50	2	0.2	27.9	14554	11.4	8.2
7	50	0.5	0.35	44.3	161892	5.1	3.6
8	50	2	0.35	56.7	51733	4.5	3.1
9	50	1.25	0.275	46.4	53257	9.1	6.1
10	50	1.25	0.275	48.2	55335	9.7	6.3
11	50	1.25	0.275	45.3	52005	8.8	5.3
12	80	0.5	0.275	46.4	133228	9.3	6.6
13	80	2	0.275	56.7	40669	6.2	4.5
14	80	1.25	0.2	31.7	26484	12.9	10.4
15	80	1.25	0.35	56.7	82875	4.7	3.6

Supplementary Table 2. Reaction parameters of 14 verification experiments for the Box-Behnken design and the corresponding reaction results of the ultrasound assisted single-phase synthesis of Ca-NDS (water). Abbreviations in this table: Reaction temperature (T), Residence time (RT), Concentration of reagents (COR), Space time yield (STY), Particle size (PS), Particle size interquartile range (PS IQR).

Experiment No.	T (°C)	RT (min)	COR (mmol mL^{-1})	Yield (%)	STY (kg $m^{-3}day^{-1}$)	PS (μm)	PS IQR (μm)
1	20	0.5	0.35	18.1	66006	5.1	3.1
2	20	1.25	0.275	43.7	50180	7.5	3.3
3	20	2	0.2	10.6	5542	11.4	8.7
4	20	2	0.35	51.8	47276	5.3	3.5
5	50	0.5	0.275	22.6	64892	6.2	4.1
6	50	1.25	0.2	16.1	13417	13.5	11.1
7	50	1.25	0.35	54.9	80259	4.3	2.9
8	50	2	0.275	51.3	36830	7.4	4.4
9	80	0.5	0.2	24.5	51118	14.6	11.2
10	80	0.5	0.35	52.3	191114	9.1	6.7
11	80	1.25	0.275	51.6	59238	7.2	4.8
12	80	2	0.2	36.3	18958	12.1	7.9
13	80	2	0.35	59.8	54627	5.4	2.5

Supplementary Table 3. Evaluation of fitting quality for linear response surface equations for space time yield (STY) by varying the number of coefficients. The selected number of terms, exhibiting a limiting gain in R^2 for increasing the number of terms, among the highest R^2 (pred), and a minimum in the RMSD for the verification points, is highlighted in bold.

Term number	R^2 (%)	R^2 (pred %)	RMSD for STY	RMSD for STY of verification points
4	79.57	51.14	19580	19300
5	93.15	75.34	11334	15258
**6**	**94.33**	**73.6**	**10311**	**13435**
7	94.63	63.19	10039	15905
8	94.82	48.28	9856	15779

Supplementary Table 4. Evaluation of fitting quality for linear response surface equations for particle size (PS) by varying the number of coefficients. The selected number of terms, exhibiting limiting gain in R^2 for increasing the number of terms, the highest R^2 (pred), and a minimum in the RMSD for the verification points, is highlighted in bold**.**

Term number	R^2 (%)	R^2 (pred %)	RMSD for PS	RMSD for PS of verification points
6	95.67	85.36	1.72	7.72
7	97.29	83.94	1.36	5.31
**8**	**98.97**	**89.67**	**0.84**	**5.13**
9	99.03	85.01	0.82	5.14

Supplementary Table 5. Evaluation of fitting quality for linear response surface equations for particle size IQR (PS IQR) by varying the number of coefficients. The selected number of terms, exhibiting limiting gain in R^2 for increasing the number of terms, the highest R^2 (pred), and a minimum in the RMSD for the verification points, is highlighted in bold.

Term number	R^2 (%)	R^2 (pred %)	RMSD for PS IQR	RMSD for PS IQR of verification points
6	95.87	86.83	1.49	4.7
**7**	**98.33**	**89.7**	**0.95**	**4.41**
8	98.74	87.7	0.82	4.41
9	98.89	83.27	0.77	4.43

The retained terms for each regression equation are given below (coefficients differ between equations as a result of independent fitting for each measured response), with coefficients in lower case *a, b, c,…* etc. and reaction parameters denoted as temperature *T*, residence time *t*, and concentration of reagents *C*:

$$STY = -a + bT + ct + dC + e(t^2) - f(Tt) - g(tC)$$

$$particle\ size = a - bT - ct - dC + e(T^2) + f(C^2) + g(Tt) + h(TC) + i(tC)$$

$$particle\ size\ IQR = a - bT - ct - dC + e(C^2) + f(Tt) + g(TC) + h(tC)$$

Supplementary Table 6. Coefficients determined from response surface fitting (equations above).

Equations	Coefficient values								
	a	b	c	d	e	f	g	h	i
STY	326885	2067	136387	1220152	16771	1140	549641	-	-
Particle size	146.1	1.133	31.72	486.6	0.002509	384	0.1678	1.9	62.7
Particle size IQR	148.2	0.6482	33.13	625.1	631	0.0989	1.6	82.7	-

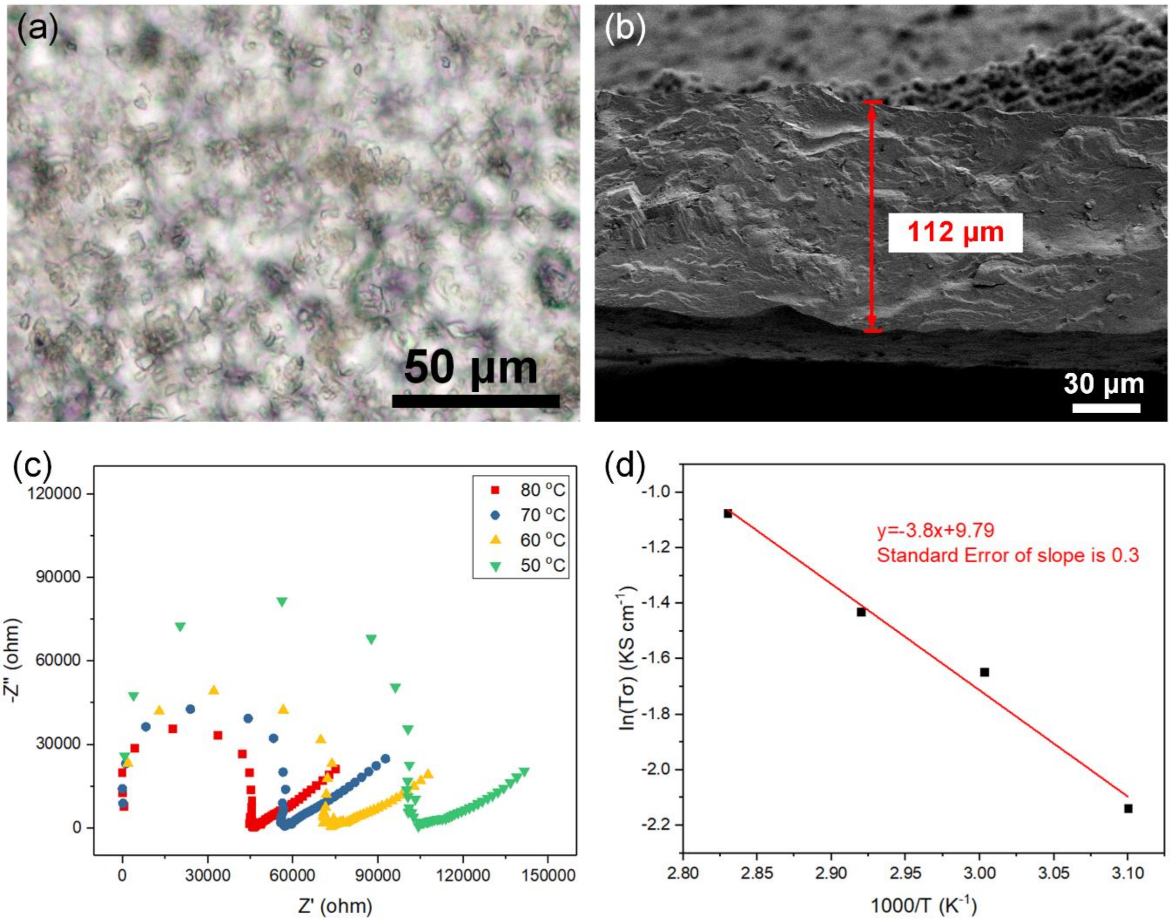

Supplementary Figure 3. (a) Light microscope image of Ca-NDS (water)-MMM; (b) Cryo-SEM micrograph of a cryo-FIB cross section of the fully hydrated (a) Ca-NDS (water); (c) EIS Nyquist plots and (d) Arrhenius plot of Ca-NDS (water)-MMM-1 between 50-80 °C and 95% RH.

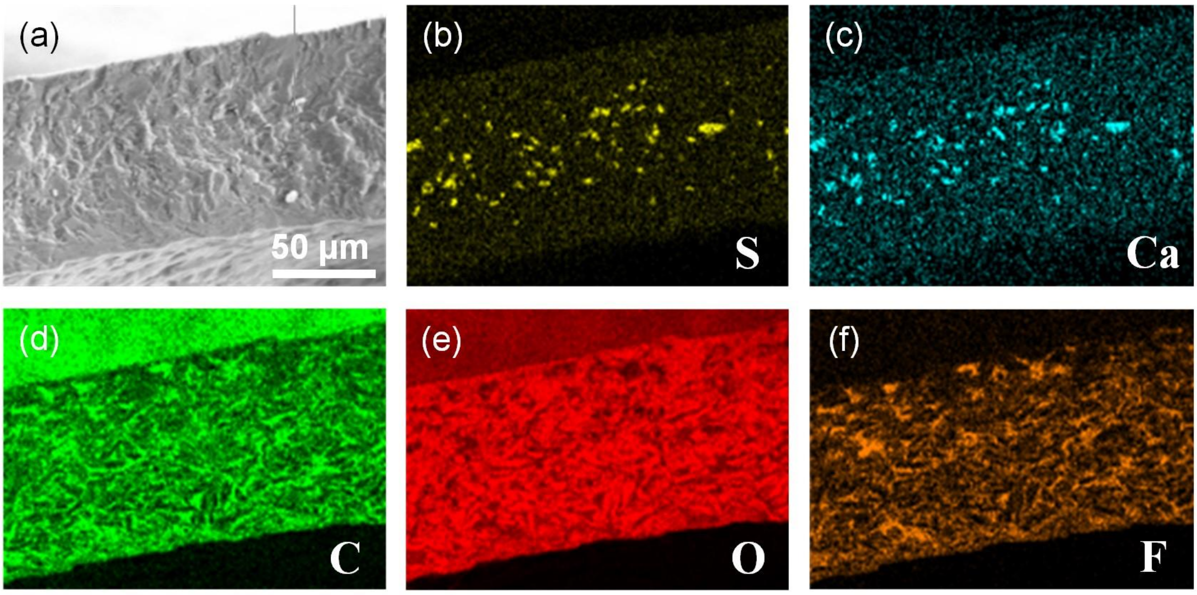

Supplementary Figure 4. (a) Cryo-SEM cross-sectional image of the fully hydrated Ca-NDS (water)-MMM-1 and SEM-EDS elemental maps of (b) sulfur, (c), calcium, (d) carbon, (e) oxygen, and (f) fluorine.

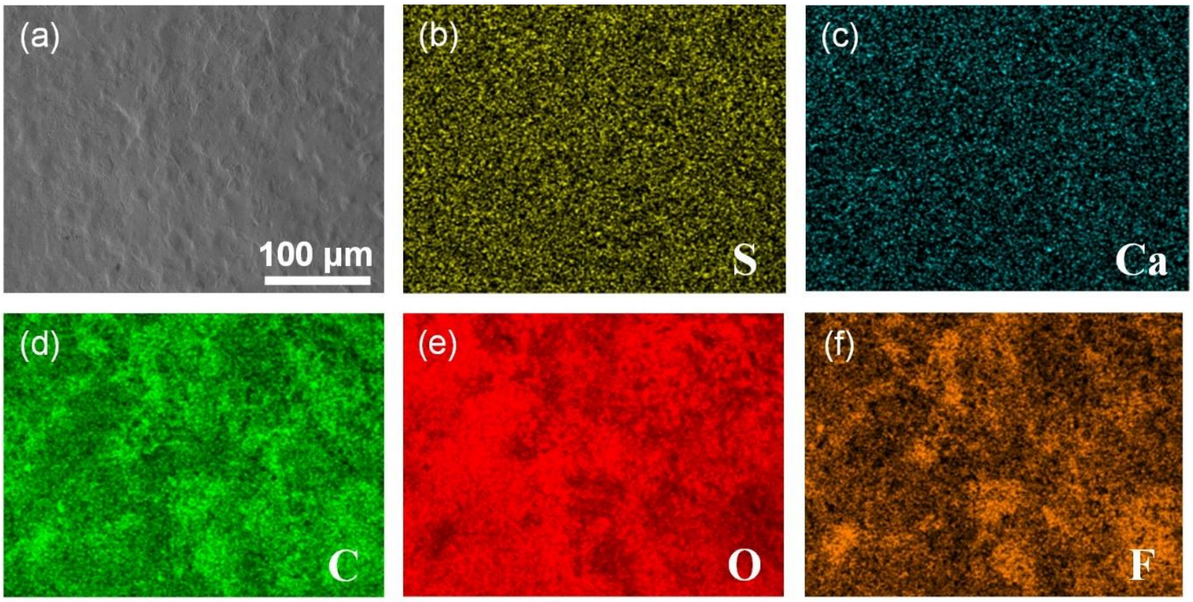

Supplementary Figure 5. (a) Cryo-SEM image of the surface of the fully hydrated Ca-NDS (water)-MMM-1 and SEM-EDS elemental maps of (b) sulfur, (c), calcium, (d) carbon, (e) oxygen, and (f) fluorine.

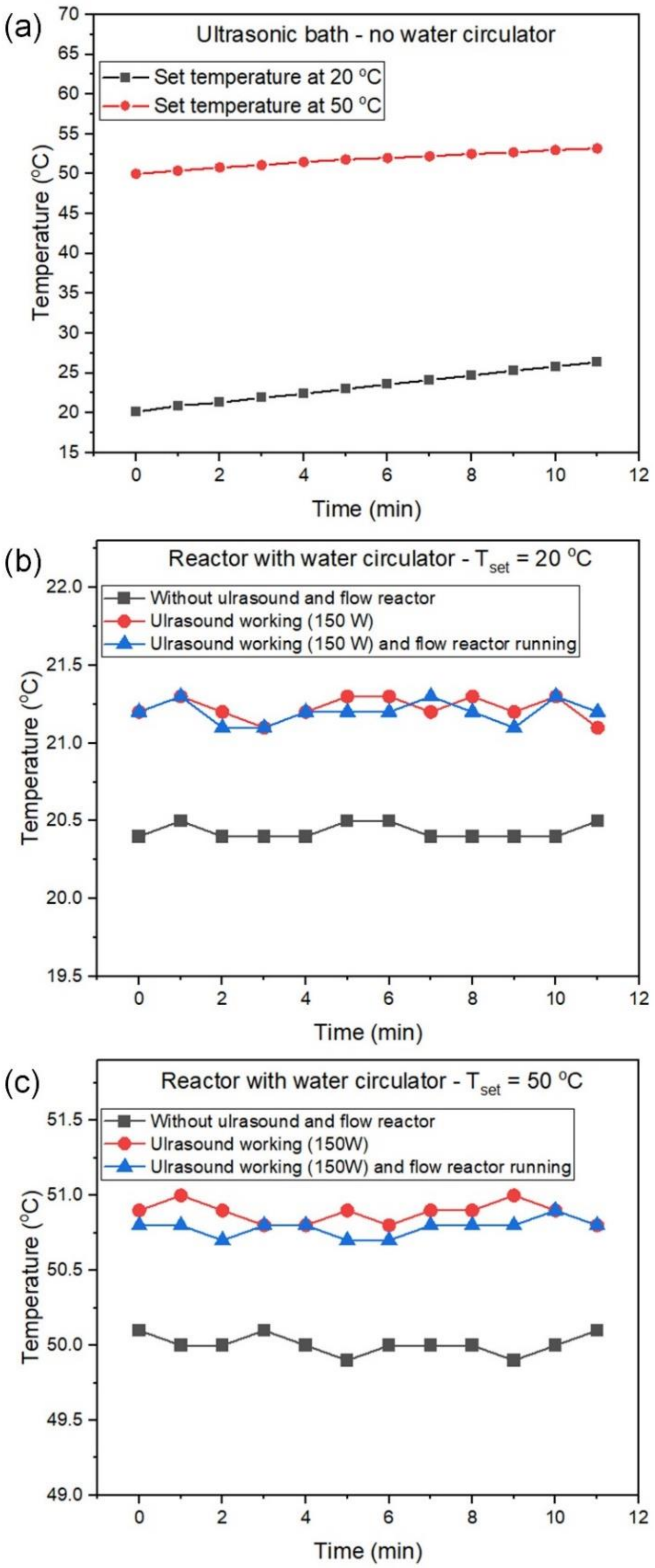

Supplementary Figure 6. (a) Temperature records of the ultrasonic bath when set at temperatures of 20 °C and 50 °C with exposure to ultrasound; temperature records of the updated reaction platform with a water circulation bath when set at temperatures of (b) 20 °C and (c) 50 °C.

Supplementary Table 7. Yield and space time yield (STY) comparison between reactions using different ultrasound powers in the single-phase synthesis of Ca-NDS (water).

Power (W)	20 °C 1.25 min 0.275 M		50 °C 0.5 min 0.275 M		50 °C 2 min 0.275 M		80 °C 1.25 min 0.275 M	
	Yield (%)	STY (kg m^{-3} day^{-1})	Yield (%)	STY (kg m^{-3} day^{-1})	Yield (%)	STY (kg m^{-3} day^{-1})	Yield (%)	STY (kg m^{-3} day^{-1})
50	32 ± 1	$3.7×10^4$ ± $1×10^3$	20 ± 2	$5.4×10^4$ ± $5×10^3$	47 ± 3	$3.4×10^4$ ± $2×10^3$	47 ± 2	$5.3×10^4$ ± $2×10^3$
100	44 ± 2	$5.0×10^4$ ± $2×10^3$	23 ± 2	$6.5×10^4$ ± $4×10^3$	51 ± 2	$3.7×10^4$ ± $2×10^3$	52 ± 2	$6.0×10^4$ ± $2×10^3$
150	47 ± 2	$5.4×10^4$ ± $2×10^3$	24 ± 2	$7.0×10^4$ ± $5×10^3$	53 ± 2	$3.8×10^4$ ± $2×10^3$	53 ± 2	$6.1×10^4$ ± $2×10^3$

Supplementary Table 8. Particle size (PS) and particle size interquartile range (PS IQR) comparison between reactions using different ultrasound powers in the single-phase synthesis of Ca-NDS (water).

Power (W)	20 °C 1.25 min 0.275 M		50 °C 0.5 min 0.275 M		50 °C 2 min 0.275 M		80 °C 1.25 min 0.275 M	
	PS (µm)	PS IQR (µm)	PS (µm)	PS IQR (µm)	PS (µm)	PS IQR (µm)	PS (µm)	PS IQR (µm)
50	16.0	7.8	14.8	6.6	11.4	7.3	9.4	6.1
100	7.5	4.3	6.2	4.1	7.4	4.4	7.2	4.8
150	6.3	3.7	5.7	2.7	6.7	3.3	6.5	3.5

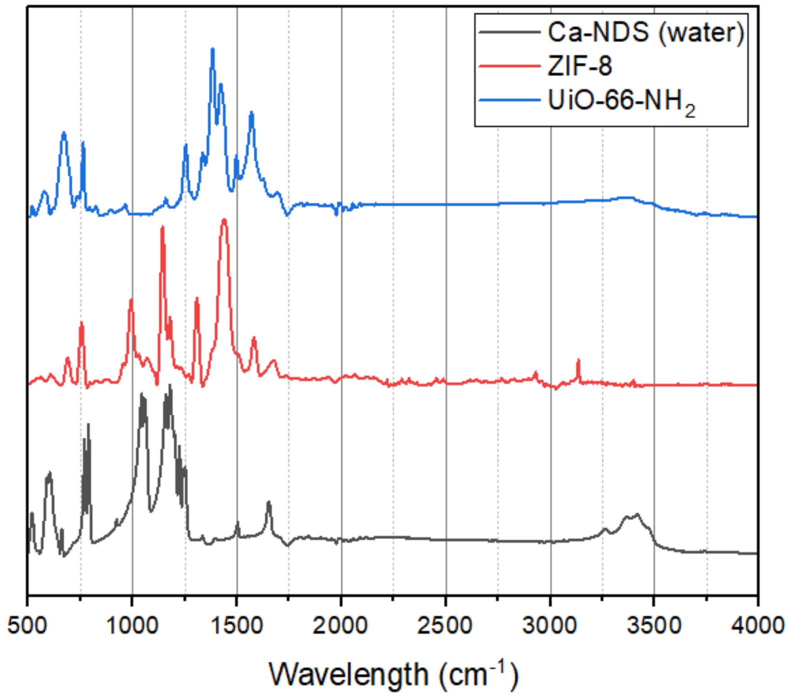

Supplementary Figure 7. ATR-FTIR spectra of Ca-NDS (water), ZIF-8, and UiO-66-NH_2 samples synthesized in two-phase flow. For Ca-NDS (water), strong bands at approximately at 530, 620, 1068 and 1190 cm^{-1} were assigned as the major peaks of the sulfonate group [2]. Multiple peaks across 1450 to 1650 cm^{-1} were attributed to the skeleton vibration of the benzene rings [3], and peaks in the range 650 to 900 cm^{-1} were assigned to the out-of-plane C-H bending vibration of the aromatic ring [4]. A wider and stronger band between 3000 to 3500 cm^{-1} was attributed to O-H band from water bound to Ca in the Ca-NDS (water) unit cell. For ZIF-8, the bands at 687 and 754 cm^{-1} in the fingerprint region were associated with out-of-plane bending of the imidazole ring, whereas peaks in the region of between 900 and 1350 cm^{-1} arose from the in-plane bending [5]. The peaks at 1581 and 1678 cm^{-1} were attributed to the bending and stretching N–H vibrations of the imidazole group [6]. Two weak peaks at 2930 and 3124 cm^{-1} are assigned to the aliphatic and aromatic C–H stretching of the imidazole, respectively [7]. For UiO-66-NH_2, the characteristic peak at 768 cm^{-1} was associated with Zr–O stretching vibration [8]. The peak at 1425 cm^{-1} was associated with the C–C vibrational bond, and the peaks at 1382 and 1572 cm^{-1} were attributed to symmetric and asymmetric C–O stretching bonds respectively, resulting from aromatic and carboxylic groups [9]. The bonding between aromatic carbon and nitrogen (C–N) could be observed at 1258 and 1338 cm^{-1}, and the peak at 1619 cm^{-1} was assigned as the bending vibration of N–H, and the wide band between 3200 and 3500 cm^{-1} were derived from the amine group [8].

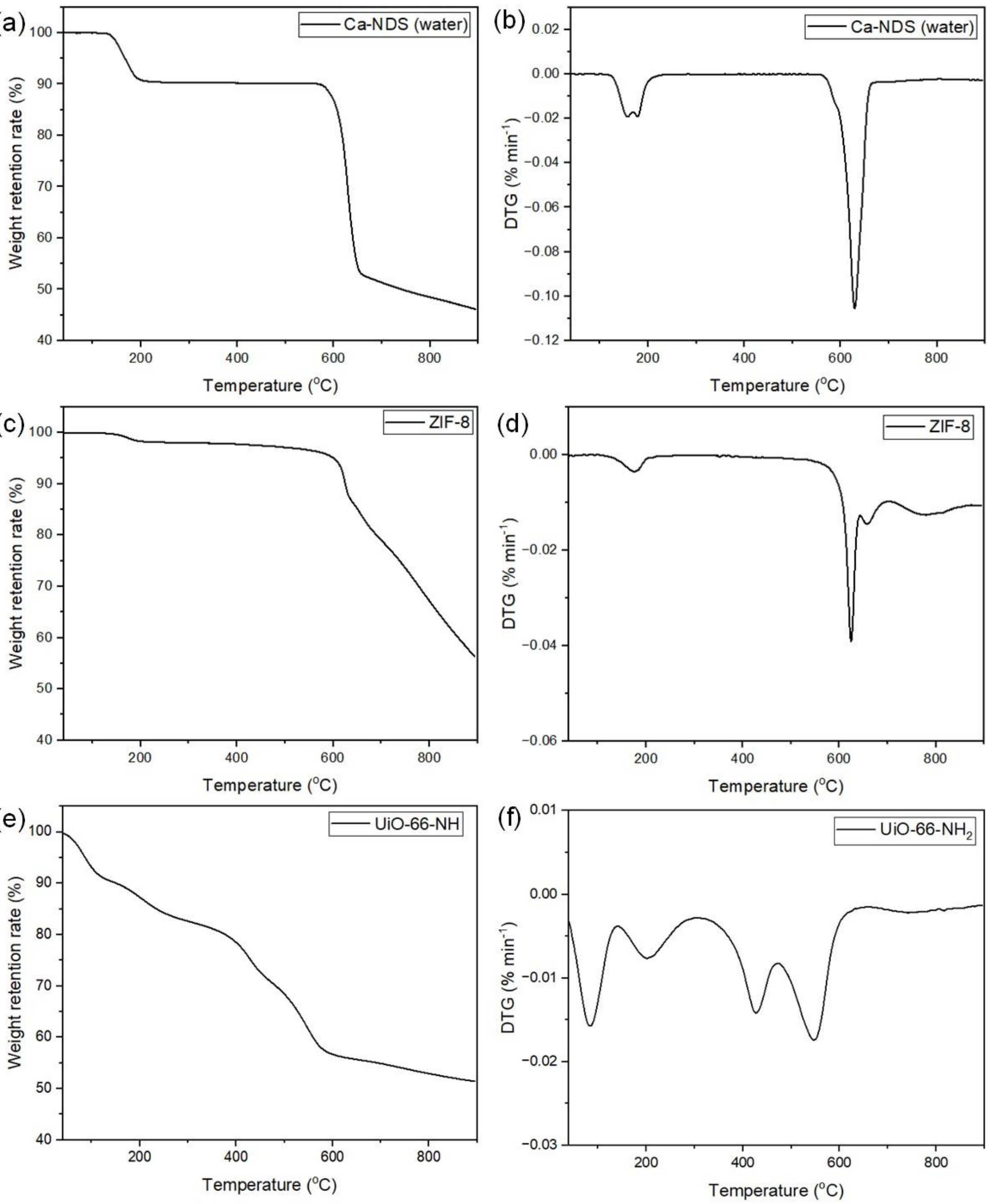

Supplementary Figure 8. TGA and the corresponding differential thermogravimetry (DTG) curves obtained on (a-b) Ca-NDS (water), (c-d) ZIF-8, and (e-f) UiO-66-NH_2 samples synthesized in two-phase flow. The TGA curve of the as-synthesized Ca-NDS (water) showed an initial mass loss from approximately 170 °C, indicating loss of coordinated water molecules, followed by NDS decomposition above 600 °C (a and b). ZIF-8 similarly showed the first weight-loss step around 170 °C corresponding to the release of the absorbed water, a sharp weight loss at approximately 620 °C was caused by structural degradation and decomposition of Hmim ligands (c and d). The TGA curve of UiO-66-NH_2 featured a mass decrease at around 90 °C caused by the loss of surface water molecules (e and f), the second small weight decrease around 200 °C corresponded to the loss of water molecules coordinated in the cage within MOFs, followed by the collapse the structure of MOF and decomposition of H_2ATA ligands [10]. All temperatures were extracted from the maxima in the first derivative of the TGA curve (the DTG curve).

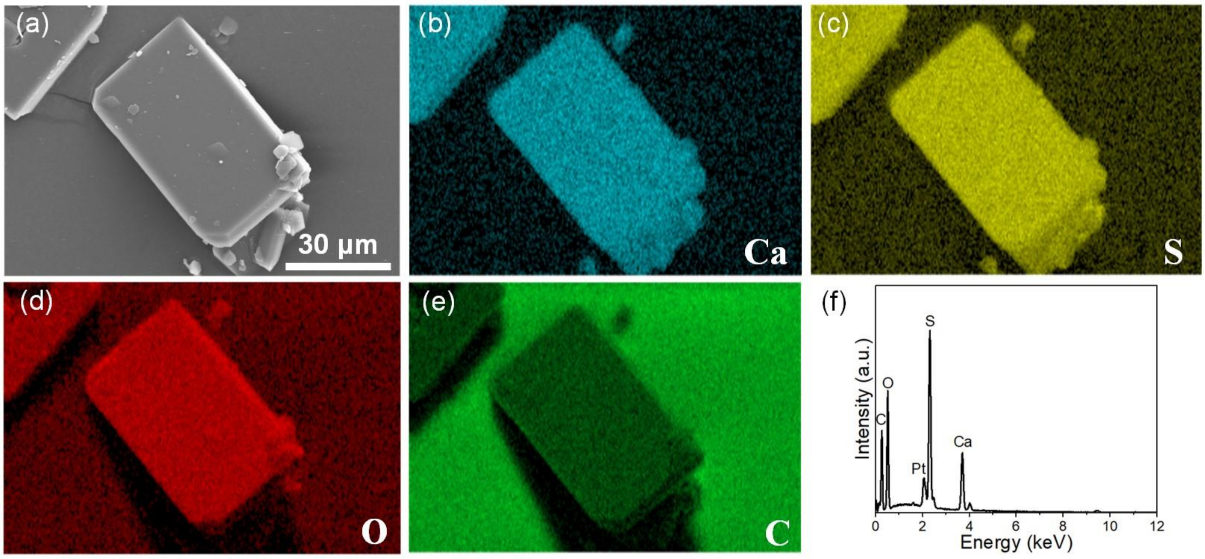

Supplementary Figure 9. (a) SEM image of Ca-NDS (water) particles synthesized in two-phase flow and SEM-EDS elemental maps of (b) calcium, (c) sulfur, (d) oxygen, and (e) carbon. (f) Integrated EDS spectrum from the entire field of view.

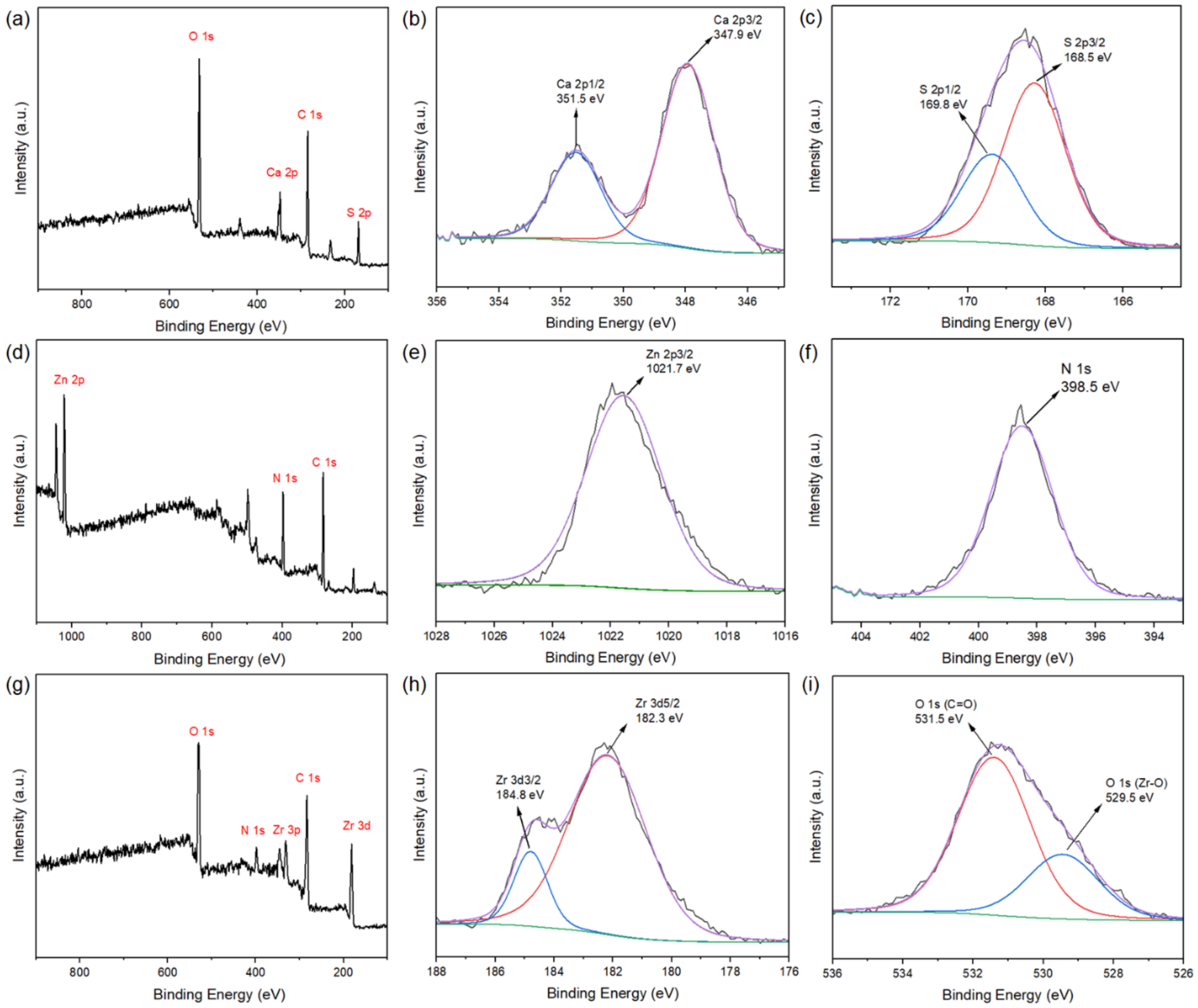

Supplementary Figure 10. XPS analyses of Ca-NDS (water), ZIF-8, and UiO-66-NH_2 synthesized in two-phase flow. (a) Survey spectra and high resolution scans of (b) the Ca 2p peak corresponding to Ca^{2+} $2p_{3/2}$ and $2p_{1/2}$ [11] in the Ca-NDS (water) structure and (c) the S 2p peak for Ca-NDS (water) showing $2p_{3/2}$ and $2p_{1/2}$ peaks at 168.5 and 169.8 eV. These S 2p features were attributed to the SO_3^{2-} group [12] in the Ca-NDS (water) structure. (d) Survey spectra and high resolution scans of (e) the Zn^{2+} $2p_{3/2}$ peak (1021.7 eV [13]) and (f) the N 1s for ZIF-8 with a peak at 398.5 eV attributed to the imidazole groups in ZIF-8 [14]. (g) Survey spectra and (h) high resolution scan of Zr 3d peak showing two peaks at 182.3 and 184.8 eV assigned to Zr^{4+} $3d_{5/2}$ and $3d_{3/2}$, respectively [15]. These features indicate the formation of Zr-O bonds in the metal cluster of UiO-66-NH_2. (i) High resolution scan of O 1s peak for UiO-66-NH_2 showing peaks at binding energies of 531.5 and 529.5 eV, attributed to C=O [16] and Zr-O bonds [15, 17], respectively.

Supplementary Table 9. Elemental composition by SEM-EDS analysis (atomic %) of Ca-NDS (water) synthesized in two-phase flow. The quantification was carried out using the Cliff-Lorimer method with manufacturer-supplied k-factors. The theoretical elemental ratios for the Ca-NDS (water) unit cell [1] are included for comparison.

Elements	C %	O %	S %	Ca %	Ratio of Ca/(Ca+S)
Ca-NDS (water)	51.1	32.8	10.8	5.3	0.33
Unit cell formula Ca-NDS (DMF)	47.6	38.1	9.5	4.8	0.34

Supplementary Table 10. Elemental composition by XPS (atomic%) of Ca-NDS (water) synthesized in two-phase flow. The theoretical elemental ratios for the Ca-NDS (water) unit cell [1] are included for comparison.

Elements	C 1s %	O 1s %	S 2p %	Ca 2p %	Ratio of Ca/(Ca+S)
Ca-NDS (water)	55.6	28.2	10.8	5.4	0.33
Unit cell formula Ca-NDS (DMF)	47.6	38.1	9.5	4.8	0.34

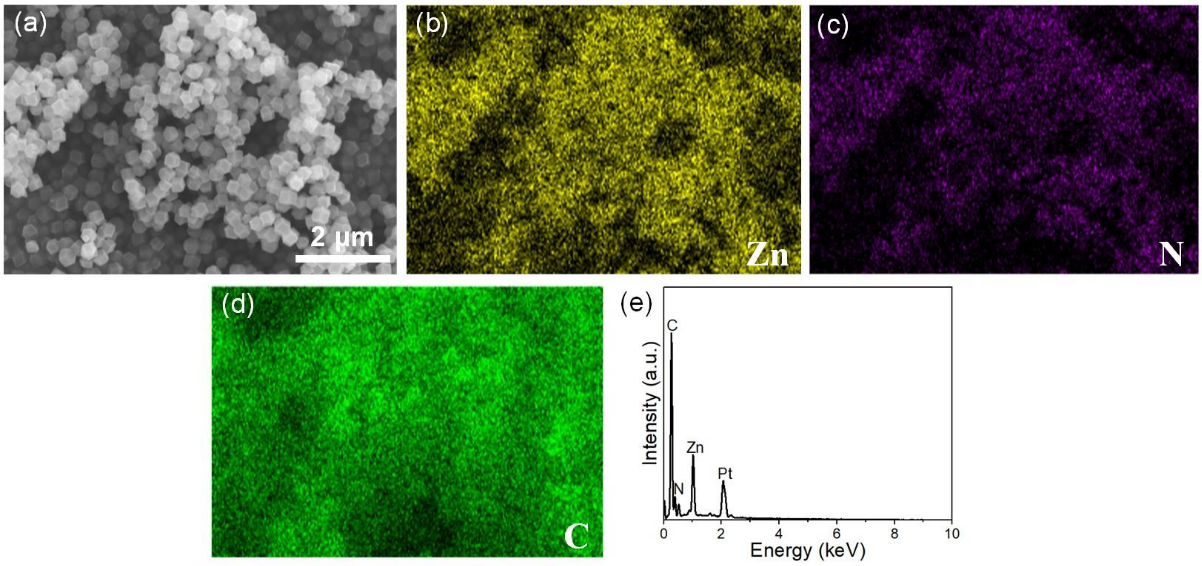

Supplementary Figure 11. (a) SEM image of ZIF-8 particles synthesized n two-phase flow and SEM-EDS elemental maps of (b) zinc, (c) nitrogen, and (d) carbon. (e) Integrated EDS spectrum from the entire field of view.

Supplementary Table 11. Elemental composition by SEM-EDS analysis (atomic %) of ZIF-8 synthesized in two-phase flow. The quantification was carried out using the Cliff-Lorimer method with manufacturer-supplied k-factors. The theoretical elemental ratios for the ZIF-8 unit cell [18] are included for comparison.

Elements	C %	N %	Zn %	Ratio of Zn/(Zn+N)
ZIF-8	74.2	21.4	4.4	0.17
Unit cell formula ZIF-8	61.5	30.8	7.7	0.2

Supplementary Table 12. Elemental composition by XPS (atomic%) of ZIF-8 synthesized in two-phase flow. The theoretical elemental ratios for the ZIF-8 unit cell [18] are included for comparison.

Elements	C 1s %	N 1s %	Zn 2p %	Ratio of Zn/(Zn+N)
ZIF-8	70.6	25.3	4.1	0.14
Unit cell formula ZIF-8	61.5	30.8	7.7	0.2

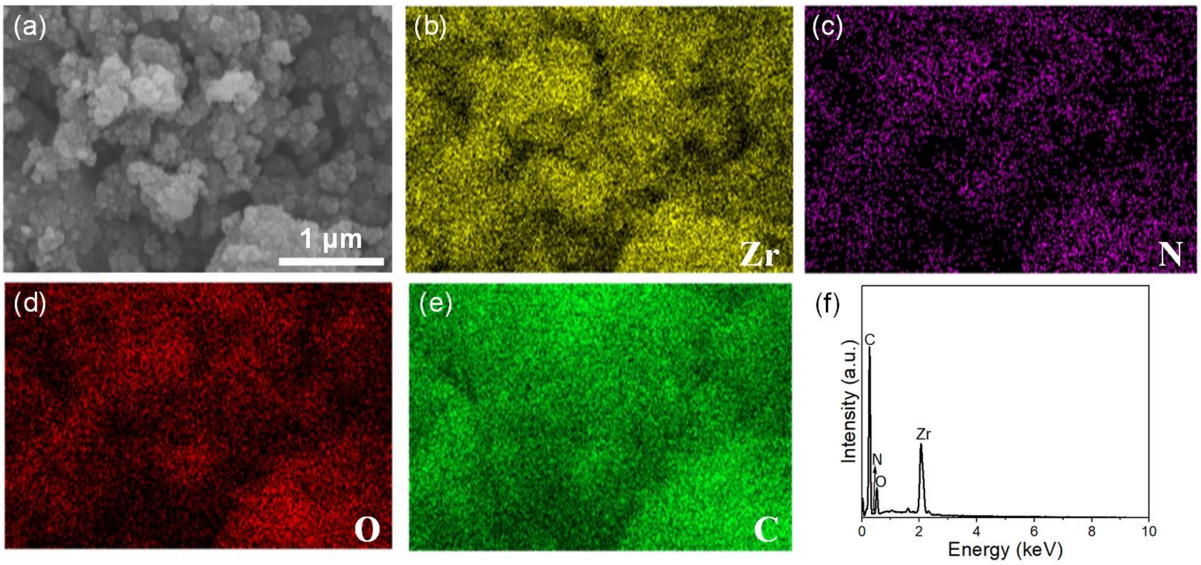

Supplementary Figure 12. (a) SEM image of UiO-66-NH_2 particles flow synthesized in two-phase flow and SEM-EDS elemental maps of (b) zirconium, (c) nitrogen, (d) oxygen, and (e) carbon. (f) Integrated EDS spectrum from the entire field of view.

Supplementary Table 13. Elemental composition by SEM-EDS analysis (atomic %) of UiO-66-NH_2 synthesized in two-phase flow. The quantification was carried out using the Cliff-Lorimer method with manufacturer-supplied k-factors. The theoretical elemental ratios for the UiO-66-NH_2 unit cell [19] are included for comparison.

Elements	C %	O %	N %	Zr %	Ratio of Zr/(Zr+O)	Ratio of Zr/(Zr+N)
UiO-66-NH_2	85.7	11.6	1.0	1.8	0.13	0.64
Unit cell formula UiO-66-NH_2	43.6	44.4	5.5	6.6	0.13	0.55

Supplementary Table 14. Elemental composition by XPS (atomic%) of UiO-66-NH_2 synthesized in two-phase flow. The theoretical elemental ratios for the UiO-66-NH_2 unit cell [19] are included for comparison.

Elements	C 1s %	O 1s %	N 1s %	Zr 3d %	Ratio of Zr/(Zr+O)	Ratio of Zr/(Zr+N)
NH_2-UIO-66	60.6	27.7	5.7	6.0	0.18	0.51
Unit cell formula NH_2-UiO-66	43.6	44.4	5.5	6.6	0.13	0.55

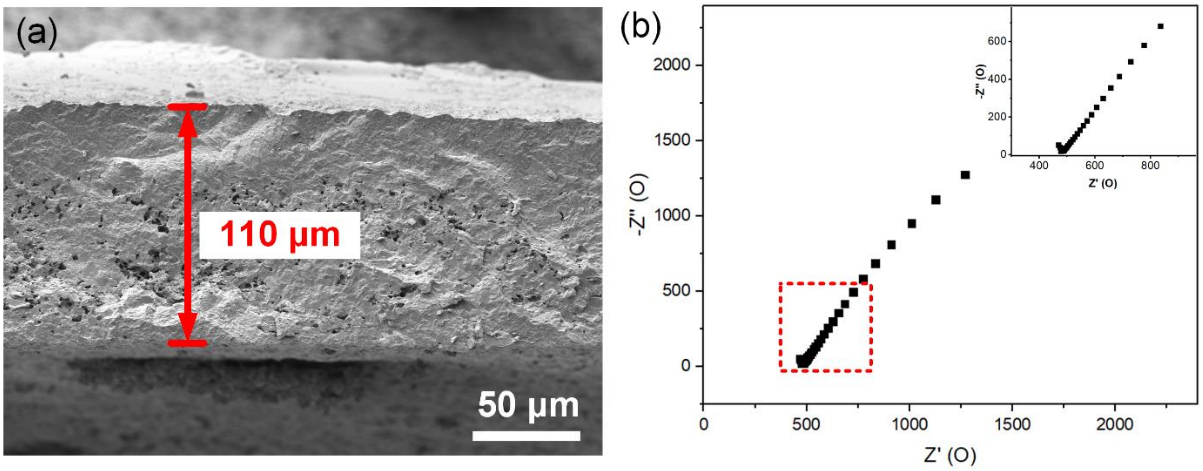

Supplementary Figure 13. (a) Cryo-SEM micrograph of a cross section of the fully hydrated Ca-NDS (water)-MMM-2; and (b) EIS Nyquist plot for Ca-NDS (water) based pellet measured at 80 °C and 95% RH.

Supplementary Table 15. Density, resistance, and proton conductivities of Ca-NDS (water) pellets prepared from material synthesized in two-phase flow. Uncertainties are given as one standard deviation.

Sample name	Density (g cm^{-3})	Calculated density (g cm^{-3})	Density as % of calculated density	Thickness (cm)	Resistance (ohm)	Proton conductivity (mS cm^{-1})
Ca-NDS (water) a	1.44	1.746	82.63	0.145	485	1.53
Ca-NDS (water) b	1.52		86.94	0.121	432	1.43
AVG of Ca-NDS (water)	1.48 ± 0.04		85 ± 2	--	--	1.48 ± 0.05

Supplementary Table 16. BET surface area and pore volume of ZIF-8 synthesized by batch, ultrasound-assisted batch, and two-phase flow in ultrasonic bath approaches.

Synthesis methods and median particle size	Batch (600 nm)	Batch in ultrasonic bath (500 nm)	Two-phase flow in ultrasonic bath (330 nm)
BET surface area (m^2 g^{-1})	1587	1735	1886
Pore volume (cm^3 g^{-1})	0.644	0.670	0.721

Supplementary Table 17. BET surface area and pore volume of UiO-66-NH_2 synthesized by batch, ultrasound-assisted batch, and two-phase flow in ultrasonic bath approaches.

Synthesis methods and particle size	Batch	Batch in ultrasonic bath	Two-phase flow in ultrasonic bath
BET surface area (m^2 g^{-1})	743	795	787
Pore volume (cm^3 g^{-1})	0.663	0.720	0.756

Supplementary Table 18. STY and E factor of Ca-NDS (water) synthesized by batch, ultrasound-assisted batch, and ultrasound-assisted two-phase flow methods at 50 °C.

Reaction parameters	30 min batch	2 min batch (100 W)	0.75 min two-phase flow 50 W	0.75 min two-phase flow 100 W	0.75 min two-phase flow 150 W
Yield %	36 ± 2	38 ± 2	28 ± 2	32 ± 2	36 ± 1
STY (kg m^{-3} day^{-1})	1.7×10^3 $\pm 1\times10^2$	1.4×10^4 $\pm 1\times10^3$	2.7×10^4 $\pm 2\times10^3$	3.1×10^4 $\pm 1\times10^3$	3.4×10^4 $\pm 1\times10^3$
Production rate (g h^{-1})	1.06 ± 0.06	8.8 ± 0.6	10.0 ± 0.7	11.5 ± 0.4	12.6 ± 0.4
E factor	18.73	17.69	24.36	21.19	18.73
E factor excluding water	1.78	1.63	2.57	2.13	1.78

Note: E factor $= \frac{Total\ mass\ of\ waste}{Mass\ of\ final\ product}$

Supplementary Table 19. STY and E factor of ZIF-8 synthesized by batch, ultrasound-assisted batch, and ultrasound-assisted two-phase flow methods at 50 °C.

Reaction parameters	180 min batch	5 min batch (100 W)	0.75 min two-phase flow 50 W	0.75 min two-phase flow 100 W	0.75 min two-phase flow 150 W
Yield %	41 ± 2	25 ± 2	21 ± 1	25.2 ± 0.9	27 ± 1
STY (kg m^{-3} day^{-1})	$3.1\times10^1 \pm 6$	$7.2\times10^2 \pm 5\times10^1$	$3.1\times10^3 \pm 2\times10^2$	$3.7\times10^3 \pm 1\times10^2$	$4.0\times10^3 \pm 2\times10^2$
Production rate (g h^{-1})	0.02 ± 0.01	0.45 ± 0.03	1.15 ± 0.07	1.37 ± 0.04	1.49 ± 0.07
E factor	240.29	390.06	465.05	390.06	364.83
E factor excluding water	27.52	45.23	54.10	45.23	42.25

Supplementary Table 20. STY, E factor and solvent intensity of UiO-66-NH_2 synthesized by batch, ultrasound-assisted batch, and ultrasound-assisted two-phase flow methods at 50 °C.

Reaction parameters	180 min batch	5 min batch (100 W)	1 min two-phase flow 50 W	1 min two-phase flow 100 W	1 min two-phase flow 150 W
Yield %	19.6 ± 0.7	10.3 ± 0.4	6.6 ± 0.4	7.9 ± 0.6	8.7 ± 0.5
STY (kg m^{-3} day^{-1})	$4.6\times10^{1} \pm 2$	$8.7\times10^{2} \pm 4\times10^{1}$	$1.5\times10^{3} \pm 1\times10^{2}$	$1.8\times10^{3} \pm 1\times10^{2}$	$2.0\times10^{3} \pm 1\times10^{2}$
Production rate (g h^{-1})	0.029 ± 0.001	0.54 ± 0.03	0.56 ± 0.04	0.67 ± 0.04	0.74 ± 0.04
E factor	172.58	328.81	512.99	429.18	390.85
E factor excluding water	32.23	62.14	97.40	81.36	74.02
Solvent intensity	23.00	43.71	68.12	57.01	51.93

Note: solvent intensity includes the use of glacial acetic acid in this case.

$$\text{Solvent intensity} = \frac{Total\ mass\ of\ solvent\ used\ excluding\ water}{Mass\ of\ final\ product}$$

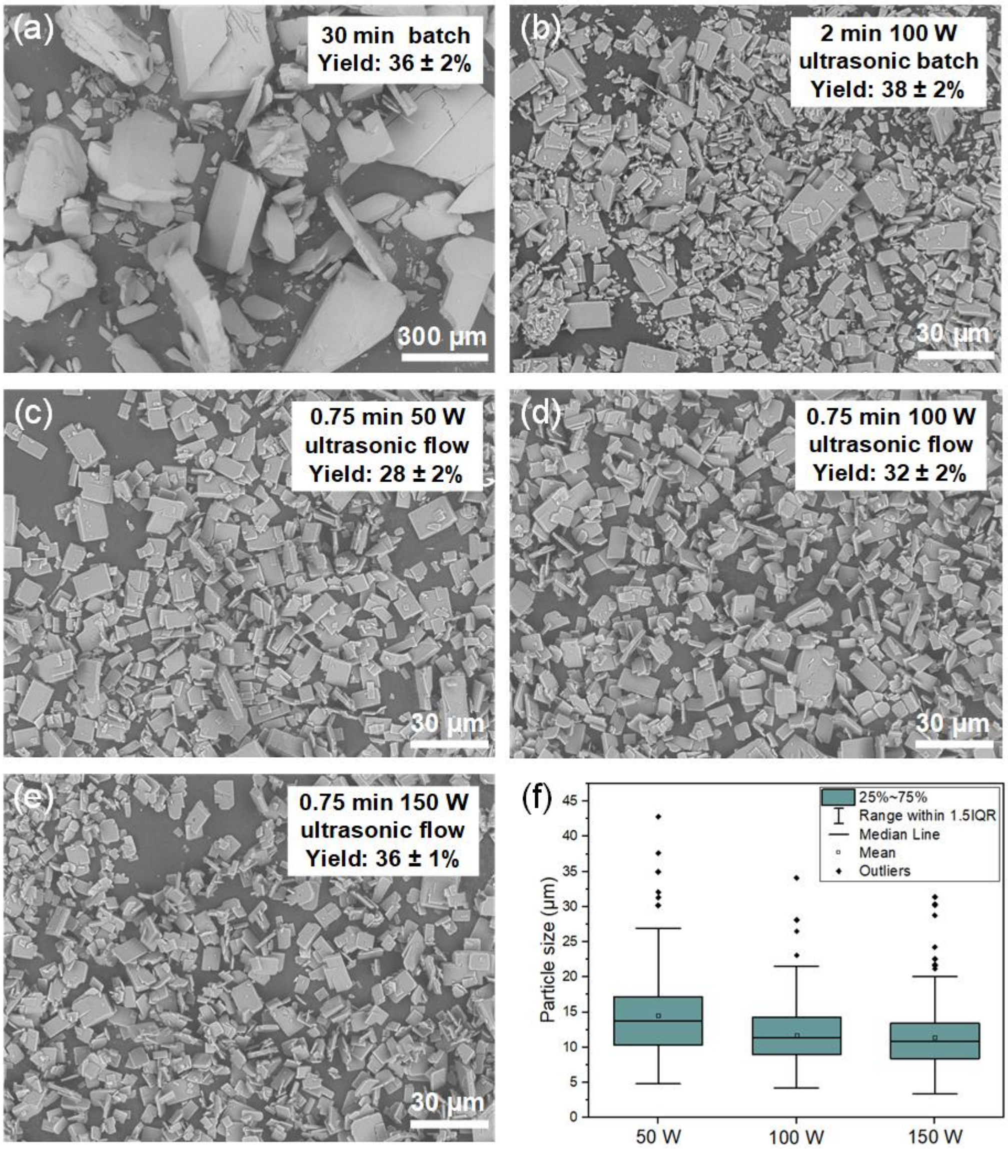

Supplementary Figure 14. SEM images of Ca-NDS (water) particles synthesized in (a) batch with a reaction time of 30 min; (b) ultrasonic batch with a reaction time of 2 min; (c)-(e) two-phase flow (0.75 min or 45 s residence time) using an ultrasonic power of (c) 50 W, (d) 100 W, and (e) 150 W. (f) Box charts of particle size for the samples made in two-phase flow shown in (c)-(e).

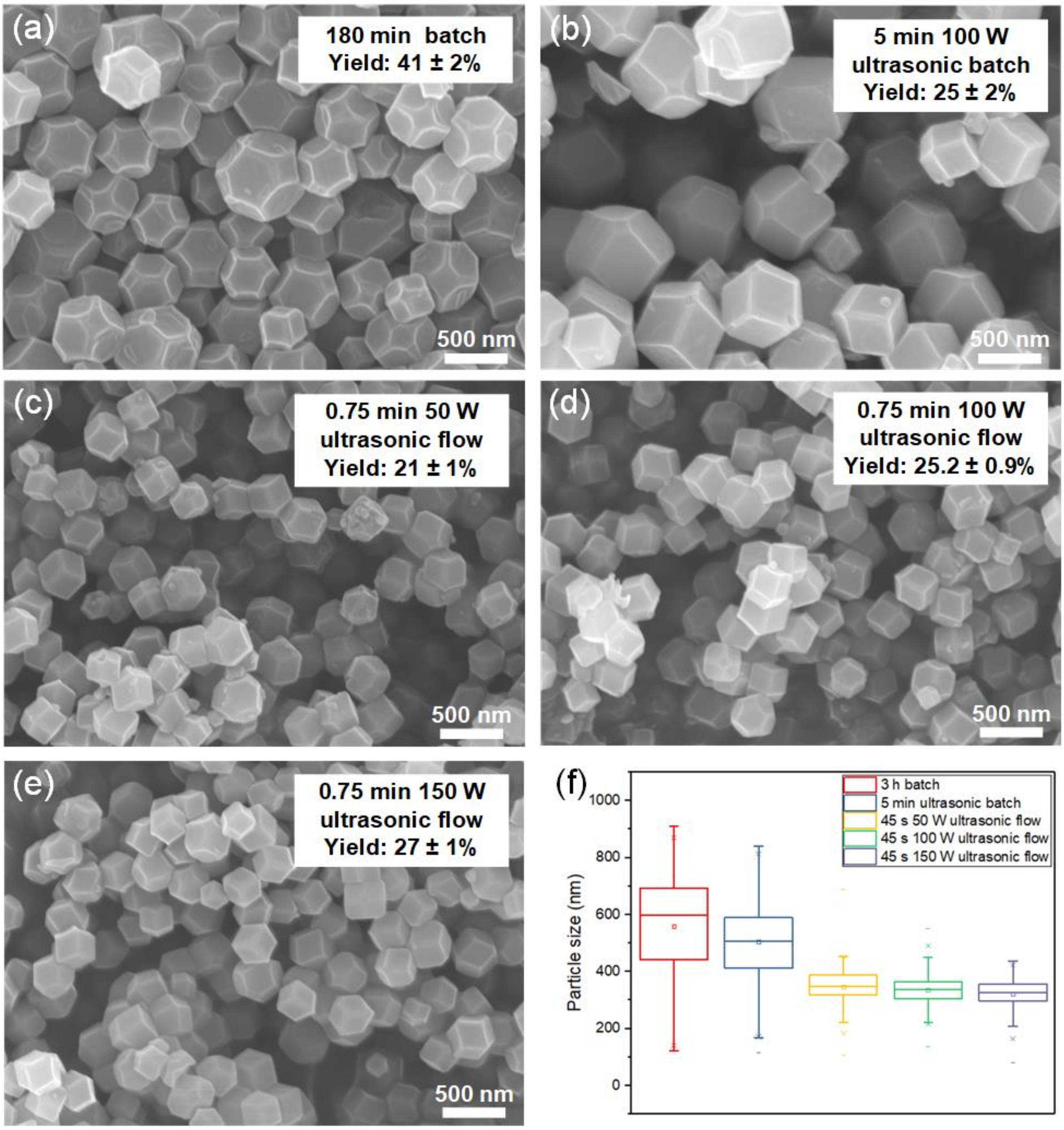

Supplementary Figure 15. SEM images of ZIF-8 particles synthesized in: (a) batch with a reaction time of 180 min (3 h); (b) ultrasonic batch with a reaction time of 5 min; (c)-(e) two-phase flow (0.75 min or 45 s residence time) using ultrasonic powers of (c) 50 W, (d) 100 W, and (e) 150 W. (f) Box charts of particle size of the samples shown in (a)-(e).

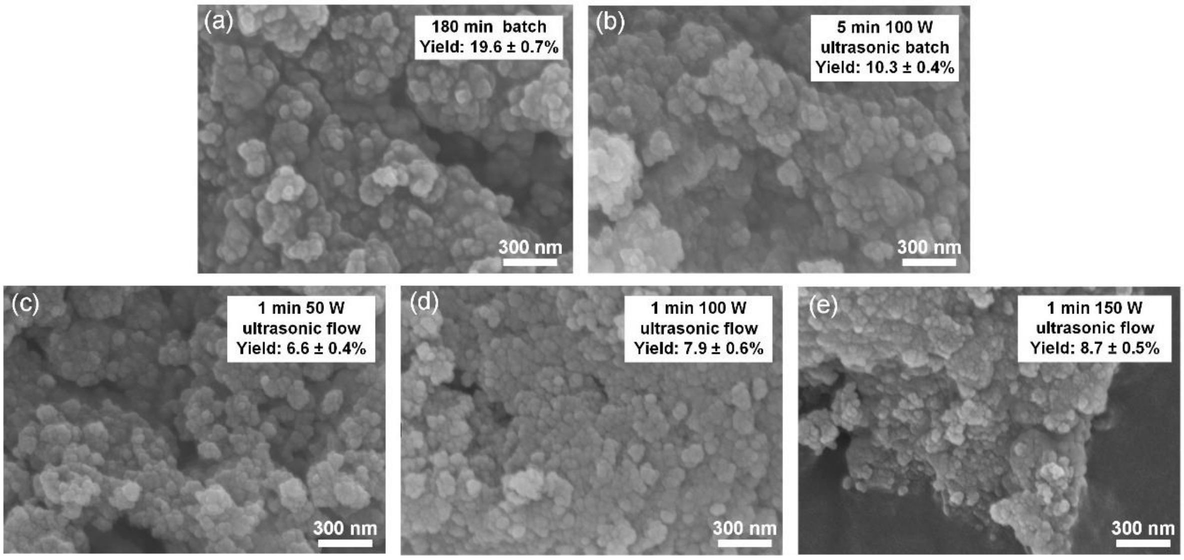

Supplementary Figure 16. SEM images of UiO-66-NH_2 samples synthesized in: (a) batch with a reaction time of 180 min (3 h); (b) ultrasonic batch within 5 min; (c)-(e) two-phase flow (1 min residence time) using ultrasonic powers of (c) 50 W, (d) 100 W, and (e) 150 W.

Supplementary Table 21. Comparison of surface areas, yields, space time yields (STYs), production rates, and surface area production rate (SAPR) for aqueous ZIF-8 syntheses reported in the literature and this work. RT denotes room temperature. Note: Yields are generally higher at longer reaction times (e.g. in hydrothermal batch syntheses) where reactions have progressed further to completion.

Solvent	Temp (°C)	Synthesis method	BET surface area (m^2 g^{-1})	Yield (%)	STY (kg m^{-3} day^{-1})	Production rate (g h^{-1})	SAPR (m^2 m^{-3} day^{-1})	Ref.
water	25	Hydro-thermal batch	1126	90	8	0.24	9.0×10^6	20
water	RT	Hydro-thermal batch	1520	97.5	6	0.6	9.1×10^6	21
water	RT	Hydro-thermal batch	1472	90	244	2.03	3.6×10^8	22
water	RT	Hydro-thermal batch	1398.7	84.5	930	0.39	1.3×10^9	23
water	RT	Hydro-thermal batch	1173	80	2337	0.86	2.7×10^9	24
water	RT	AC electrokinetic assisted continuous flow	1305	49 ± 2	83623 ± 3000	0.001 ± 0.0001	1.1×10^{11}	25
water	RT	Continuous flow	1844	--	10132	1.34	1.9×10^{10}	26
water + NH_4OH	RT	Continuous flow	1800	--	11625	27	2.1×10^{10}	27
MeOH + NH_3	RT	Continuous flow	1770	54	210000	26.67	3.7×10^{11}	28
water	50	Ultrasound-assisted two-phase flow	1886	27.1 ± 1	4000 ± 200	1.49 ± 0.07	7.5×10^9 ± 3.6×10^8	This work

Note: the specific surface area can vary depending on the calculation method, references in this table are from the main text.

Supplementary Table 22. Comparison of surface areas, yields, space time yields (STYs), production rates, and surface area production rate (SAPR) for aqueous UiO-66-NH_2 syntheses reported in the literature and this work. RT denotes room temperature. Note: Yields are generally higher at longer reaction times (e.g. in hydrothermal batch syntheses) where reactions have progressed further to completion.

Solvent	Temp (°C)	Synthesis method	BET surface area (m^2 g^{-1})	Yield (%)	STY (kg m^{-3} day^{-1})	Production rate (g h^{-1})	SAPR (m^2 m^{-3} day^{-1})	Ref.
water + acetic acid	RT	Hydro-thermal batch	854	40	716	0.6	6.1×10^8	29
water + acetic acid	RT	Hydro-thermal batch	717	37	3381 ± 54	7.04 ± 0.11	2.4×10^9	30
EtOH + formic acid	110	Hydro-thermal in vacuum	831	75	20	0.04	1.7×10^7	31
DMF	RT	Electro-chemical	844	62	32	0.08	2.7×10^7	32
water + acetic acid	85	Continuous synthesis	1150	74	4346	325	5.0×10^9	33
DMF + trifluoracetic acid	175	Microwave assisted	973	84 ± 3	1450 ± 80	3 ± 0.2	1.4×10^9	34
water + acetic acid	90	Continuous flow spray-drying	840	70	10	0.01	8.4×10^6	35
water + acetic acid	50	Ultrasound-assisted two-phase flow	787	8.7 ± 0.5	2000 ± 100	0.74 ± 0.04	1.6×10^9 ± 8.3×10^7	This work

Note: the specific surface area can vary depending on the calculation method, references in this table are from the main text.

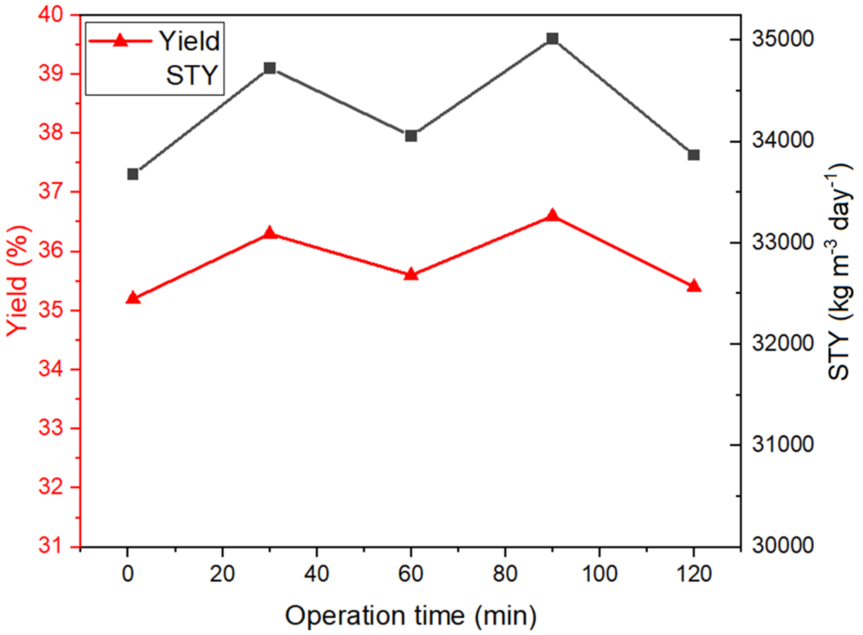

Supplementary Figure 17. Yields and STYs for continuous operation of two-phase flow synthesis of Ca-NDS (water). The reaction platform supports sustained production for over 120 min in ultrasound-assisted two-phase flow with a 0.75 min residence time and a 150 W ultrasonic power at 50 °C.

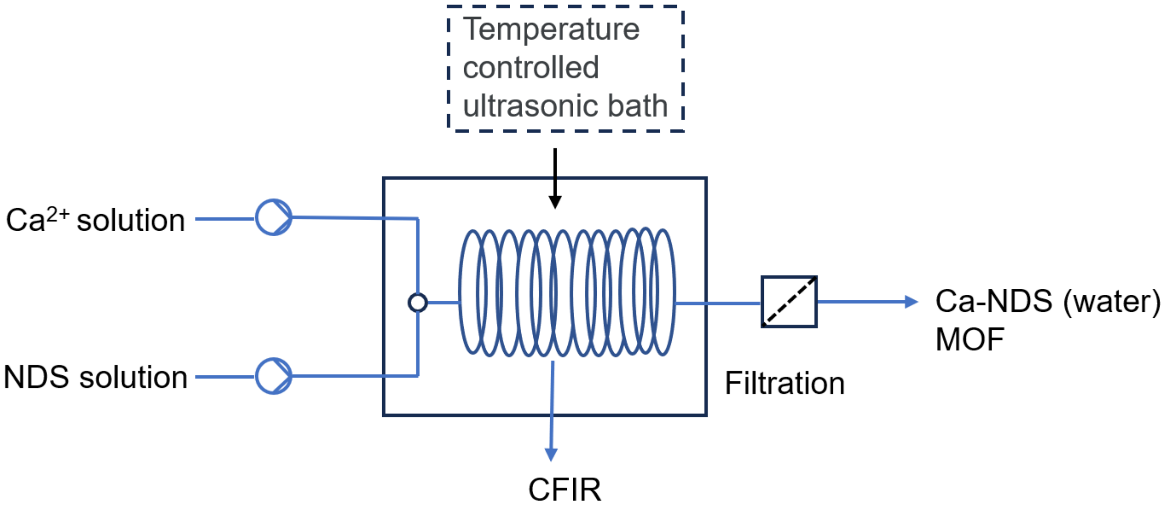

Supplementary Figure 18. Schematic diagram of the ultrasound-assisted continuous synthesis of Ca-NDS (water) in single-phase flow.

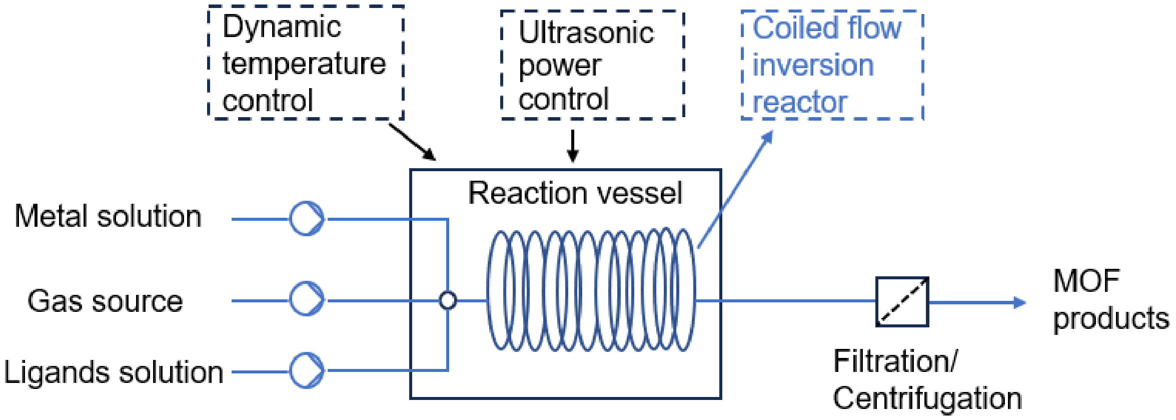

Supplementary Figure 19. A general schematic diagram of the ultrasound-assisted two-phase flow reactor for MOF synthesis.

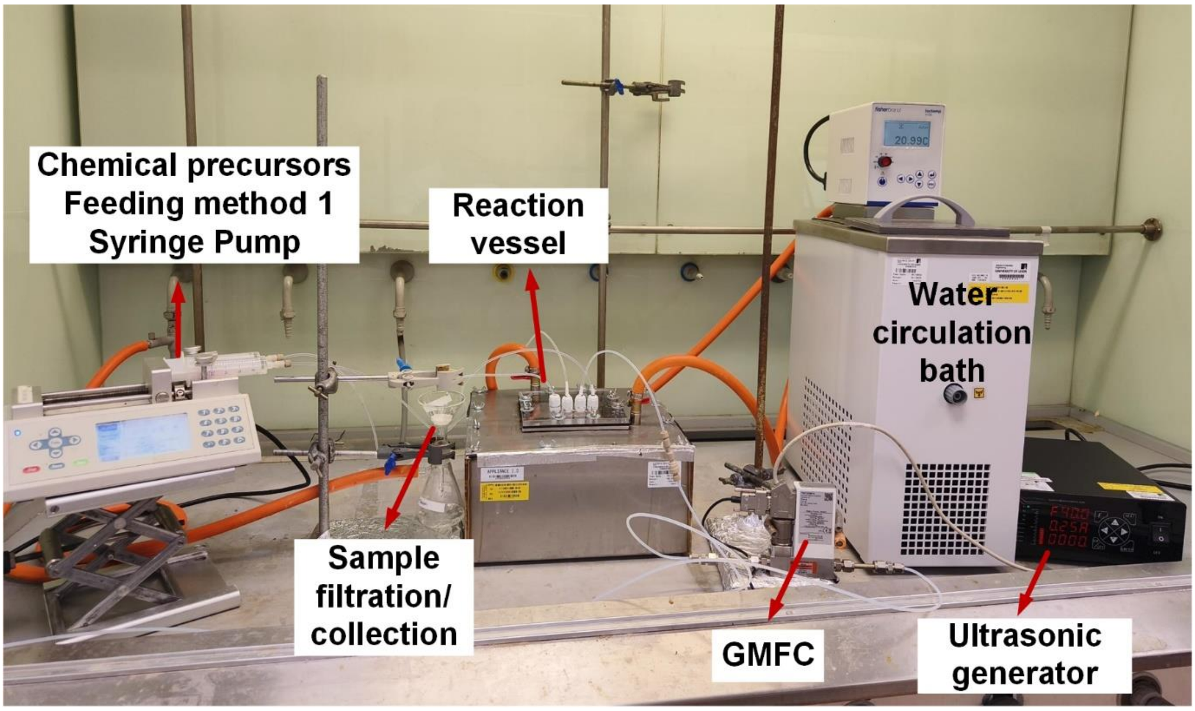

Supplementary Figure 20. A digital photo of the ultrasound-assisted two-phase flow reaction platform. GMFC denotes a gas mass flow controller.

Supplementary References:

1. Cai J, Chen CH, Liao CZ, Feng XL, Chen XM. Solid-state structures of group 1 and group 2 metal 1,5-naphthalenedisulfonates: systematic investigation of lamellar three-dimensional networks constructed by metal arenedisulfonate. *Acta Crystallogr B* **57**, 520-530 (2001).
2. Zhong L, Parker SF. Structure and vibrational spectroscopy of methanesulfonic acid. *R Soc Open Sci* **5**, 181363 (2018).
3. Staufer M*, et al.* The vibrational structure of benzene adsorbed on Si(001). *J Chem Phys* **112**, 2498-2506 (2000).
4. Ishida Y*, et al.* Time-resolved photoemission apparatus achieving sub-20-meV energy resolution and high stability. *Rev Sci Instrum* **85**, 123904 (2014).
5. Jian M, Liu B, Zhang G, Liu R, Zhang X. Adsorptive removal of arsenic from aqueous solution by zeolitic imidazolate framework-8 (ZIF-8) nanoparticles. *Colloids Surf A* **465**, 67-76 (2015).
6. He M, Yao J, Liu Q, Wang K, Chen F, Wang H. Facile synthesis of zeolitic imidazolate framework-8 from a concentrated aqueous solution. *Microporous and Mesoporous Mater* **184**, 55-60 (2014).
7. Low Z-X*, et al.* Crystal Transformation in Zeolitic-Imidazolate Framework. *Cryst Growth Des* **14**, 6589-6598 (2014).
8. Zhang X, Zhang Y, Wang T, Fan Z, Zhang G. A thin film nanocomposite membrane with pre-immobilized UiO-66-NH(2) toward enhanced nanofiltration performance. *RSC Adv* **9**, 24802-24810 (2019).
9. Wu S*, et al.* Adsorption of Cr(VI) on nano UiO-66-NH_2 MOFs in water. *Environ Technol* **39**, 1937-1948 (2018).
10. Aghajanzadeh M, Zamani M, Molavi H, Khieri Manjili H, Danafar H, Shojaei A. Preparation of Metal–Organic Frameworks UiO-66 for Adsorptive Removal of Methotrexate from Aqueous Solution. *J Inorg Organomet Polym Mater* **28**, 177-186 (2017).
11. Liu Q, Li J, Zhou Z, Xie J, Lee JY. Hydrophilic Mineral Coating of Membrane Substrate for Reducing Internal Concentration Polarization (ICP) in Forward Osmosis. *Sci Rep* **6**, 19593 (2016).
12. Liu D, Jin Z, Bi Y. Charge transmission channel construction between a MOF and rGO by means of Co–Mo–S modification. *Catal Sci Technol* **7**, 4478-4488 (2017).
13. Kumbetlioglu F, Oskay KO, Ciplak Z, Ates A. Preparation, Characterization, and Application of Metal Oxide-Doped Zeolitic Imidazolate Framework. *ACS Omega* **8**, 27650-27662 (2023).
14. Tian F*, et al.* Surface and Stability Characterization of a Nanoporous ZIF-8 Thin Film. *J Phys Chem C* **118**, 14449-14456 (2014).
15. Peñas-Garzón M*, et al.* Solar photocatalytic degradation of parabens using UiO-66-NH_2. *Sep Purif Technol* **286**, 120467 (2022).
16. Dolgov A*, et al.* Characterization of carbon contamination under ion and hot atom bombardment in a tin-plasma extreme ultraviolet light source. *Appl Surf Sci* **353**, 708-713 (2015).
17. Ren J*, et al.* Construction of efficient g-C_3N_4/NH_2-UiO-66 (Zr) heterojunction photocatalysts for wastewater purification. *Sep Purif Technol* **274**, 118973 (2021).
18. Morris W, Stevens CJ, Taylor RE, Dybowski C, Yaghi OM, Garcia-Garibay MA. NMR and X-ray Study Revealing the Rigidity of Zeolitic Imidazolate Frameworks. *J Phys Chem C* **116**, 13307-13312 (2012).
19. Trickett CA, Gagnon KJ, Lee S, Gandara F, Burgi HB, Yaghi OM. Definitive molecular

level characterization of defects in UiO-66 crystals. *AngewChem Int Ed* **54**, 11162-11167 (2015).

20. Jian*, et al.* Water-based synthesis of zeolitic imidazolate framework-8 with high morphology level at room temperature. *RSC Adv* **5**, 48433 (2015).
21. Kida K, Okita M, Fujita K, Tanaka S, Miyake Y. Formation of high crystalline ZIF-8 in an aqueous solution. *CrystEngComm* **15**, 1794 (2013).
22. Khan IU*, et al.* Structural transition from two-dimensional ZIF-L to three-dimensional ZIF-8 nanoparticles in aqueous room temperature synthesis with improved CO_2 adsorption. *Mater Charact* **136**, 407-416 (2018).
23. Prathap MUA, Gunasekaran S. Rapid and Scalable Synthesis of Zeolitic Imidazole Framework (ZIF-8) and its Use for the Detection of Trace Levels of Nitroaromatic Explosives. *Adv Sustain Syst* **2**, 1800053 (2018).
24. Pan Y, Liu Y, Zeng G, Zhao L, Lai Z. Rapid synthesis of zeolitic imidazolate framework-8 (ZIF-8) nanocrystals in an aqueous system. *Chem Commun* **47**, 2071-2073 (2011).
25. Chen S, Zhou X, Li G, Yang F. Controlled synthesis of metal-organic frameworks via AC electrokinetic mixing-assisted microfluidics: A case study of ZIF-8. *Chem Eng J* **480**, 148208 (2024).
26. Wu H*, et al.* Continuous and ultrafast MOF synthesis using droplet microfluidic nanoarchitectonics. *J Mater Chem A* **11**, 9427-9435 (2023).
27. Munn AS, Dunne PW, Tang SV, Lester EH. Large-scale continuous hydrothermal production and activation of ZIF-8. *Chem Commun* **51**, 12811-12814 (2015).
28. Polyzoidis A, Altenburg T, Schwarzer M, Loebbecke S, Kaskel S. Continuous microreactor synthesis of ZIF-8 with high space–time-yield and tunable particle size. *Chem Eng J* **283**, 971-977 (2016).
29. Pakamor I, Rousseau J, Rousseau C, Monflier E, Szilágyi Pg. An ambient-temperature aqueous synthesis of zirconium-based metal–organic frameworks. *Green Chem* **20**, (2018).
30. Huelsenbeck L*, et al.* Generalized Approach for Rapid Aqueous MOF Synthesis by Controlling Solution pH. *Cryst Growth Des* **20**, 6787-6795 (2020).
31. Zhang X, Zhang S, Ouyang G, Han R. Removal of Cr(VI) from solution using UiO-66-NH_2 prepared in a green way. *Korean J Chem Eng* **39**, 1839-1849 (2022).
32. Wei JZ*, et al.* Rapid and Low-Cost Electrochemical Synthesis of UiO-66-NH_2 with Enhanced Fluorescence Detection Performance. *Inorg Chem* **58**, 6742-6747 (2019).
33. Reinsch H, Waitschat S, Chavan SM, Lillerud KP, Stock N. A Facile "Green" Route for Scalable Batch Production and Continuous Synthesis of Zirconium MOFs. *Eur J Inorg Chem* **2016**, 4490-4498 (2016).
34. Guerrero RM*, et al.* Scaling-Up Microwave-Assisted Synthesis of Highly Defective Pd@UiO-66-NH_2 Catalysts for Selective Olefin Hydrogenation under Ambient Conditions. *ACS Appl Mater Interfaces* **16**, 24108-24121 (2024).
35. Avci-Camur C*, et al.* Aqueous production of spherical Zr-MOF beads via continuous-flow spray-drying. *Green Chem* **20**, 873-878 (2018).
