# Supplementary material for: Ultrasound-assisted continuous aqueous synthesis of sulfonate, imidazolate, and carboxylate MOFs with high space time yield
Source: Commun Chem. 2025 May 16;8:154. doi: 10.1038/s42004-025-01548-5 (PMC12084381; doi:10.1038/s42004-025-01548-5)
Supplement: Supplementary file 1 — Transparent peer review file [file 42004_2025_1548_MOESM1_ESM.pdf]

This file contains all reviewer reports in order by version, followed by all author rebuttals in order by version.

This manuscript has been previously reviewed at another journal. This document only contains information relating to versions considered at Communications Chemistry.

nature portfolio

Peer Review File

Ultrasound-assisted continuous aqueous synthesis of sulfonate, imidazolate, and carboxylate MOFs with high space time yield

Corresponding Author: Dr Sean Collins

Version 0:

Reviewer comments:

Reviewer #1

(Remarks to the Author)
The authors did a relevant job in revising the article, which is now, in the present reviewer's opinion, suitable for publication.

Open Access This Peer Review File is licensed under a Creative Commons Attribution 4.0 International License, which permits use, sharing, adaptation, distribution and reproduction in any medium or format, as long as you give appropriate credit to the original author(s) and the source, provide a link to the Creative Commons license, and indicate if changes were made.
In cases where reviewers are anonymous, credit should be given to 'Anonymous Referee' and the source.
The images or other third party material in this Peer Review File are included in the article's Creative Commons license, unless indicated otherwise in a credit line to the material. If material is not included in the article's Creative Commons license and your intended use is not permitted by statutory regulation or exceeds the permitted use, you will need to obtain permission directly from the copyright holder.
To view a copy of this license, visit https://creativecommons.org/licenses/by/4.0/

Response to reviewer comments

The reviewer comments are presented in blue italics to offset from responses in black text.

The authors did a relevant job in revising the article, which is now, in the present reviewer's opinion, suitable for publication.

We thank the reviewer for these supportive comments on the revisions and recommendation for publication.
